# Root Microbiome and Metabolome Traits Associated with Improved Post-Harvest Root Storage for Sugar Beet Breeding Lines Under Southern Idaho Conditions

**DOI:** 10.3390/ijms252312681

**Published:** 2024-11-26

**Authors:** Rajtilak Majumdar, Shyam L. Kandel, Carl A. Strausbaugh, Anuradha Singh, Suresh Pokhrel, Malick Bill

**Affiliations:** 1 Northwest Irrigation and Soils Research Laboratory (NWISRL), United States Department of Agriculture (USDA)-Agricultural Research Service (ARS), Kimberly, ID 83341, USA; pokhrelsuresh@gmail.com (S.P.); malick.bill@ndsu.edu (M.B.); 2 Sugar Beet Research, USDA-ARS, Fargo, ND 58102, USA; shyam.kandel@usda.gov; 3 Food Animal Metabolism Research, USDA-ARS, Fargo, ND 58102, USA; anuradha.singh@usda.gov; 4 Department of Plant Pathology, North Dakota State University, Fargo, ND 58108, USA

**Keywords:** sugar beet, post-harvest storage resistance, root microbiome, bacterial marker, fungal marker, storage loss, metabolome

## Abstract

Post-harvest storage loss in sugar beets due to root rot and respiration can cause >20% sugar loss. Breeding strategies focused on factors contributing to improved post-harvest storage quality are of great importance to prevent losses. Using 16S rRNA and ITS sequencing and sugar beet mutational breeding lines with high disease resistance (R), along with a susceptible (S) commercial cultivar, the role of root microbiome and metabolome in storage performance was investigated. The R lines in general showed higher abundances of bacterial phyla, *Patescibacteria* at the M time point, and *Cyanobacteria* and *Desulfobacterota* at the L time point. Amongst fungal phyla, *Basidiomycota* (including *Athelia*) and *Ascomycota* were predominant in diseased samples. Linear discriminant analysis Effect Size (LEfSe) identified bacterial taxa such as *Micrococcales*, *Micrococcaceae*, *Bacilli*, *Glutamicibacter, Nesterenkonia*, and *Paenarthrobacter* as putative biomarkers associated with resistance in the R lines. Further functional enrichment analysis showed a higher abundance of bacteria, such as those related to the super pathway of pyrimidine deoxyribonucleoside degradation, L-tryptophan biosynthesis at M and L, and fungi, such as those associated with the biosynthesis of L-iditol 2-dehydrogenase at L in the R lines. Metabolome analysis of the roots revealed higher enrichment of pathways associated with arginine, proline, alanine, aspartate, and glutamate metabolism at M, in addition to beta-alanine and butanoate metabolism at L in the R lines. Correlation analysis between the microbiome and metabolites indicated that the root’s biochemical composition, such as the presence of nitrogen-containing secondary metabolites, may regulate relative abundances of key microbial candidates contributing to better post-harvest storage.

## 1. Introduction

Sugar beet (*Beta vulgaris* L.) is an important crop in the mid and western parts of the United States (U.S.), as ~55% of the total sugar produced in the U.S. is obtained from this crop. Following harvest, sugar beet roots are typically stored for long periods of time in outdoor piles or storage buildings as factories are unable to process the whole crop at harvest [1]. In Idaho, about two-thirds of the crop is typically stored for some period of time. Roots are stored for an average of 60 to 70 days, and in some cases for 160 days or more [2]. Storing sugar beet roots for long periods of time under ambient conditions can be challenging due to adverse weather conditions and microbial growth on the roots [1,3]. In Idaho, the average annual loss recorded between 2010 and 2012 was USD 6.40/ton of roots harvested, and an estimated 4.8 to 5.8 million tons of roots were harvested annually during this period, resulting in losses of millions of dollars [1,2,4]. For areas with colder winter temperatures in the U.S., such as Minnesota and North Dakota, roots in both outdoor and indoor piles are usually frozen solid by mid-December when ambient temperatures average <−10 °C, which stabilizes the roots for long-term storage [1,5,6]. In other areas of the U.S., such as Colorado, Idaho, Michigan, and Montana, where roots are stored under ambient conditions, only roots near the pile surface freeze since temperatures are either not cold enough or fluctuate too much to maintain the whole pile in a frozen state [1,6]. These factors, along with freeze–thaw cycles, promote microbial growth [7]. Several methods, including physical control practices such as tarping, ventilation, and stripping the outer meter of sugar beet roots from the pile surface, have been investigated to reduce storage losses [5,8,9]. Besides physical methods, chemical treatments using fungicides, such as Propulse and Stadium, have had some success; however, none of the fungicides are currently labeled for this use [2,10,11,12,13,14,15]. Despite these physical and chemical methods managing to reduce sucrose losses to some extent, the financial losses in storage are still substantially high and additional management options are needed. Identifying sources of sugar beet host resistance to rot, and use of cultivars with resistance to storage rot, can alleviate losses, although the mechanisms of such resistance are not always fully understood [16,17,18,19,20].

Plants have co-evolved with their innate microbiome to better cope with biotic and abiotic stresses [21,22,23]. Bacteria are the most abundant microbial community and constitute most of the host microbiome in both plants and animals [24]. Microbiome composition in plant tissues is not only critical in maintaining optimum plant health but is also highly important in resistance against diverse pathogens including fungi, bacteria, and viruses [24,25,26]. The beneficial role of host plant-associated bacteria against pathogens has been demonstrated in several studies and this depends upon their presence/abundance in tissues that are infected by the pathogens. As an example, the seed-endophytic bacterium, *Sphingomonas melonis*, was found only in the blight-resistant rice cultivar, and artificial inoculation of the disease-susceptible rice cultivars using the pure strain resulted in the disease-resistant phenotype [27]. This was attributed to the production of anthranilic acid (by *S. melonis*), which interferes with the protein, RpoS, involved in virulence factor biosynthesis by the seedborne pathogen, *Burkholderia plantarii*. Microbiome assembly in disease-resistant germplasms in the absence of pathogens and further restructuring of the microbiome under diseased conditions will improve our understanding of host plant resistance mechanisms. This will aid in the development of future mitigation strategies to minimize future losses in agricultural production in an eco-friendly manner.

Microbiome-related work in sugar beet has primarily focused on the identification of microbes putatively associated with resistance and/or susceptibility, predominantly against fungal pathogens related to post-harvest storage. The role of the microbiome has been investigated by comparing the rhizosphere microbiome of maritima (the ancestor of beet crops), *Beta vulgaris* ssp., and modern sugar beets, *Beta vulgaris* L. [28], field performance and disease resistance [29], roots collected from outdoor storage piles [30], genotypic differences in post-harvest storage quality and disease resistance [31], etc. Other factors affecting the post-harvest storability of sugar beet roots have been attributed to root cell anatomy, cell wall integrity, respiration and carbohydrate metabolism, metabolites such as carbohydrates/amino acids/organic acids, etc. [32,33].

Changes in the root microbiome over time are associated with changes in the root metabolome. Their contributions to resistance or susceptibility to diseases under prolonged storage conditions are poorly understood. Whether specific root metabolites may regulate the relative abundances of key microbial candidates that play roles in disease resistance is not fully understood. Storage conditions also greatly vary depending upon indoor vs. outdoor storage, changes in temperature and relative humidity, geographical locations, the prevalence of specific storage-related pathogens in specific geographical areas, etc. In this study, we used five sugar beet genotypes, including three mutational breeding lines [KSG2 (KEMS06), KSG3 (KEMS08), and KSG4 (KEMS08-600)] and a breeding line from a Polish background with high sucrose content [KSG6 (KPS25)] developed in Kimberly; ID (USDA-ARS), which were earlier demonstrated to possess significant resistance against post-harvest diseases [34,35,36], along with a susceptible commercial cultivar. The overall goal was to investigate how the root microbiome and root metabolome cooperate to improve post-harvest storability of sugar beet roots under southern Idaho conditions. We analyzed these changes in the roots at two different time points of storage, mid (M) and late (L). Our results indicate that sugar beet genotype-specific root metabolites may influence the relative abundance of potentially beneficial microbes, contributing to higher resistance against storage pathogens.

## 2. Results

Sugar beet root samples were collected at mid-storage (M) and late-storage (L) time points to investigate changes in root microbiome (bacterial and fungal) and metabolome relating to disease resistance during prolonged indoor post-harvest storage. After quality control, an average of ~84,000 raw tags/sample and ~78,000 valid tags/sample were mapped to a 16S rRNA database [37] and received zero-radius operational taxonomic units (zOTUs) (Table S1). For ITS sequencing, an average of ~386,000 raw tags/sample and ~377,000 valid tags/sample were obtained (Table S2). The rarefaction curves (Figure S1) obtained from 16S, along with ITS sequencing, revealed that the observed_OTUs identified in the samples obtained at the M and L time points rarefied/plateaued at a specific number of sequences for all samples. This indicated that deeper sequencing would not have resulted in increasing the number of OTUs obtained for both bacterial and fungal microbiomes in the samples. Sugar beet mutational breeding lines, along with another breeding line of Polish origin developed in Kimberly (ID) that showed improved resistance (R) to post-harvest diseases, were compared to a susceptible (S) commercial cultivar.

### 2.1. Bacterial and Fungal Phyla and Genera Showed Significant Changes Between the Resistant and Susceptible Lines and with Storage Times

**Figure 1:**
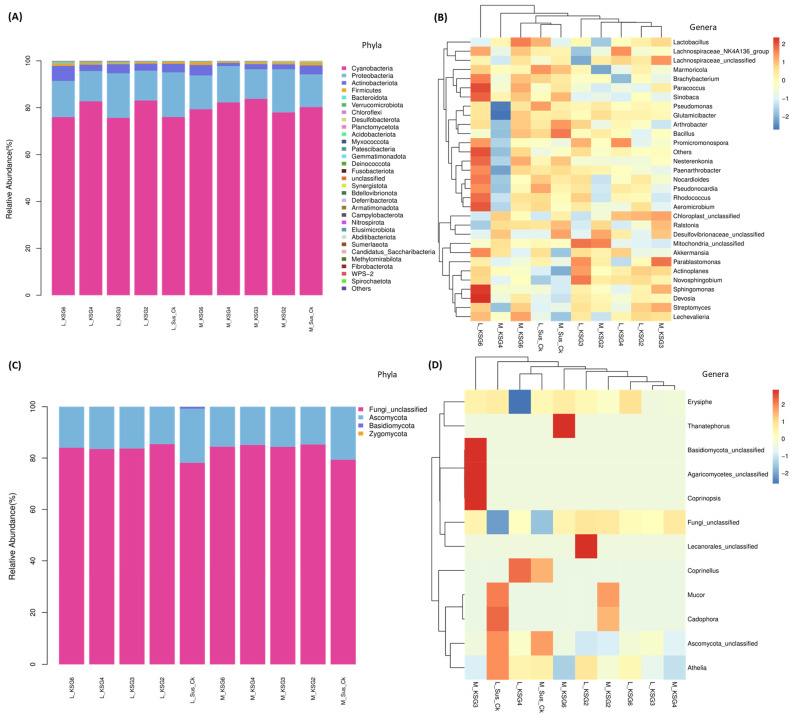
Sugar beet genotypes relatively resistant to post-harvest storage diseases showed differences in the abundance of bacterial and fungal phyla and genera compared to the susceptible genotype. Mean relative abundance of (**A**) bacterial phyla; (**B**) bacterial genera; (**C**) fungal phyla; and (**D**) fungal genera in sugar beet roots at mid and late post-harvest storage stages. Susceptible genotype: Sus_Ck; resistant genotypes: KSG2, KSG3, KSG4, and KSG6; M: mid time point; L: late time point. The data are mean ± standard error of 4 biological replicates, each replicate consists of tissues harvested from 2 sugar beet roots. The heat maps (**B**,**D**) are plotted using z-score values of the species abundance. ‘0’ means the abundance is at the mean value. Red color means the species abundance is higher than the mean, and blue color means that the species abundance is lower than the mean. Blue to red transition means abundance of the species is transitioning from ‘lower than mean’ to ‘higher than mean’.

A total of 31 bacterial phyla were detected in the roots of R and S lines across all samples and only six phyla showed a minimum abundance of 0.1% and/or higher across a majority of the samples (Figure 1A; Table S3). At both M and L storage time points, bacteria belonging to the phylum *Cyanobacteria* were predominant (76–84%) in the roots of R and S lines, followed by *Proteobacteria* (13–19%) and *Actinobacteria* (1–6%). In general, bacterial phyla whose abundances were 0.1–0.9% included *Firmicutes* and *Desulfobacterota*, both of which were lower in the R lines, and *Patescibacteria*, which were higher in the R lines at the M time point (Figure S2A). *Cyanobacteria* were predominant at the L time point and were higher in the R lines, KSG2 and KSG4, in comparison to the S line (Figure S2B).

At the M time point, examples of bacterial genera that primarily exhibited higher relative abundances in the R lines included *Erythrobacteraceae*_unclassified, *HT002*, *Desulfovibrio*, etc. Bacterial genera that were relatively more prevalent in the S line included *Desulfovibrionaceae*, *Bacillus*, *Methyloversatilis*, *Neisseria*, *Kocuria*, *Terribacillus*, etc. (Figure 1B and Figure S3). At the L time point, examples of bacterial taxa that showed higher relative abundances primarily in the R lines included *Erythrobacteraceae*, *Vicinamibacteraceae*, *Desulfovibrio*, etc. Bacterial genera with higher relative abundances in the S line at the L time point included *Lactobacillus*, *Ralstonia*, *Marmoricola*, etc. (Figure 1B, Table S4).

A total of three predominant fungal phyla could be resolved in the roots of R and S lines across all samples and only one phylum (*Ascomycota*) showed an abundance of >10% in this study (Figure 1C; Table S5). At the M time point, fungal phyla detected in our samples included *Ascomycota* (15–29%), *Basidiomycota* (<0.1%), and unresolved fungal phyla named as fungi_unclassified (70–85%). No significant differences in fungal phyla were observed between the R and S lines at this time point. At the L time point, the fungal phyla detected included *Ascomycota* (14–21%), *Zygomycota* (<0.1%), *Basidiomycota* (<1%), and fungi_unclassified (78–85%). The phylum, *Zygomycota*, showed higher relative abundance in the S line in comparison to the R lines at this time point (Figure S4A). The fungal genera, *Mucor*, showed higher relative abundance in the S line at the L time point vs. R lines (Figure 1D and Figure S4B).

### 2.2. Bacterial and Fungal Diversity in the Roots Varied Depending upon Genotype and Storage Stages

#### 2.2.1. Alpha Diversity

The alpha diversity between the R and S lines at both M and L time points was quantified using observed_OTUs and Shannon index analyses. In general, no major difference in alpha diversity was observed among samples except for a few specific cases. A significant difference in bacterial diversity was observed between KSG4 and Sus_Ck at the M time point, as estimated through observed_OTUs and Chao1 analyses (Table 1A). The KSG6 line showed significant differences in fungal diversity (vs. Sus_Ck) at M, as estimated through observed_OTUs and Shannon analyses (Table 1B).

#### 2.2.2. Beta Diversity

**Figure 2:**
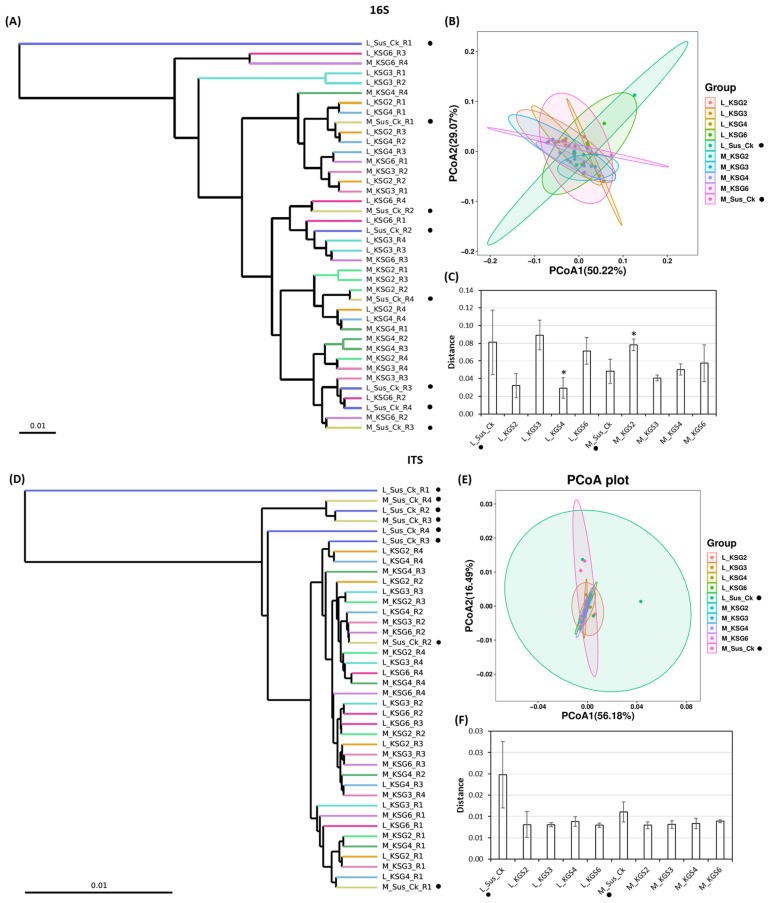
Beta diversity of bacteria and fungi in the resistant (R: KSG2, 3, 4, and 6) and susceptible (S: Sus_Ck) sugar beet genotypes at mid (M) and late (L) storage time points. (**A**) Cluster dendrogram of bacterial diversity; (**B**) principal coordinate analysis (PCoA) of bacterial diversity; (**C**) comparison of weighted UniFrac distances between S and R genotypes (16S); (**D**) cluster dendrogram of fungal diversity; (**E**) principal coordinate analysis (PCoA) of fungal diversity; and (**F**) comparison of weighted UniFrac distances between S and R genotypes (ITS). The data are mean ± standard error of 4 replicates (each replicate consists of 2 sugar beet roots); * *p* < 0.05. Solid dark circle next to the treatment represents the susceptible genotype.

To estimate microbial community structure (between-sample diversity) considering their relative abundance and phylogenetic relationships in specific samples, beta diversity analysis was performed. Cluster dendrograms and Principal Coordinate Analysis (PCoA) were used to compare and visualize microbial communities among samples. The cluster dendrograms in Figure 2A,C show the hierarchical relationship between different treatments. The PCoA variation (16S sequencing) observed between PCoA1 (50.22%) and PCoA2 (29.07%) indicated separation among treatments (Figure 2B). This variation (ITS) was relatively larger (PCoA1: 56.18%, and PCoA2: 16:49%) in the case of the fungal microbiome among treatments (Figure 2D). Comparison of weighted UniFrac distances among samples showed significant differences (*p* < 0.05) in the root bacterial community between Sus_Ck (S) and KSG2 at the M time point and Sus_Ck (S) and KSG4 at the L time point (Figure 2C). For the fungal community, weighted UniFrac distances showed significant differences (*p* < 0.05) between Sus_Ck (S) and KSG3, 4, and 6 at the L time point (Figure 2F). In general, the R and S lines were relatively clustered together for both bacteria and fungi, and a separation between samples at the M and L storage time points was observed.

**Figure 3:**
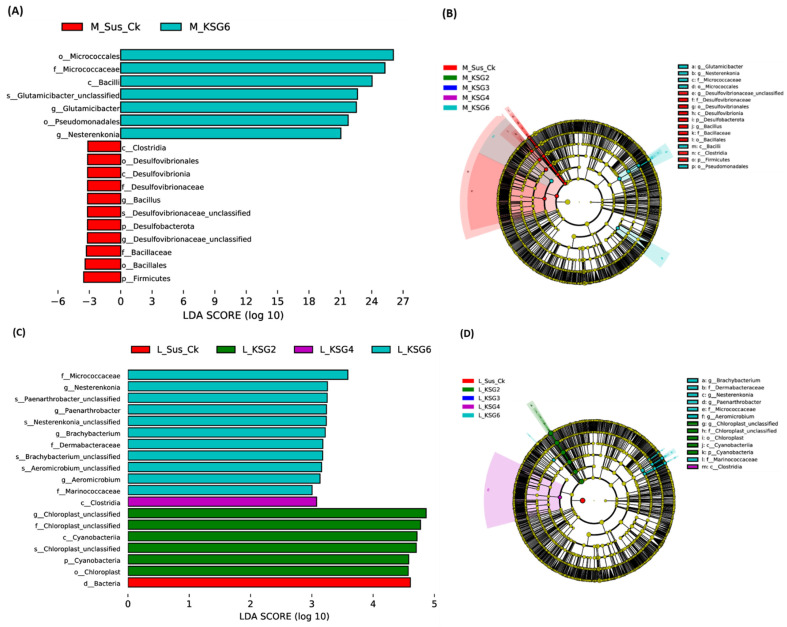
Linear discriminant analysis Effect Size (LEfSe) analysis of sugar beet genotypes at different post-harvest storage time points shows putative biomarkers associated with resistant or susceptible genotypes. (**A**) Bar plot of bacterial taxa at the mid (M) time point; (**B**) hierarchal taxonomic cladogram of bacterial taxa at the mid time point; (**C**) bar plot of bacterial taxa at the late (L) time point; and (**D**) hierarchal taxonomic cladogram of bacterial taxa at the late time point. Susceptible genotype: Sus_Ck; resistant genotypes: KSG2, KSG3, KSG4, and KSG6. Lowercase letters denote d: domain; p: phylum; c: class; o: order; f: family; g: genus.

Linear discriminant analysis Effect Size (LEfSe) was used to identify any potential biomarkers associated with the R lines used in this study. At the M time point, the LEfSe comparison between Sus_Ck and the R lines showed a putative bacterial marker only in the KSG6 line. Examples of bacterial taxa associated with KSG6 (Figure 3A,B), with high LDA scores (between 22–27), included *Micrococcales*, *Micrococcaceae*, *Bacilli*, *Glutamicibacter*, etc. At the L time point (Figure 3C,D), *Cyanobacteria* were predominant in KSG2, *Clostridia* in KSG4, and *Micrococcaceae*, *Nesterenkonia*, and *Paenarthrobacter* in KSG6. No fungal biomarkers associated with the R and S genotypes used in this study could be resolved using LefSe analysis when compared at the M and L time points.

### 2.3. Pathway Enrichment Analysis of the Microbiome Data Showed Differential Regulation in the Resistant vs. Susceptible Lines

To obtain further insights into the relationship between the relative abundance of specific bacteria or fungi in the roots of R and S lines and their putative roles in post-harvest disease resistance, functional profiling (KEGG pathway association) of both bacterial and fungal communities was performed. KEGG analysis of fungal microbiome data did not exhibit any differences between the R and S lines.

**Figure 4:**
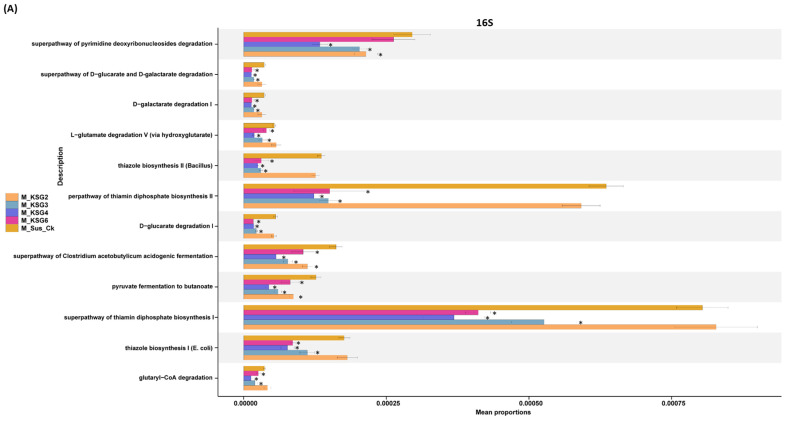

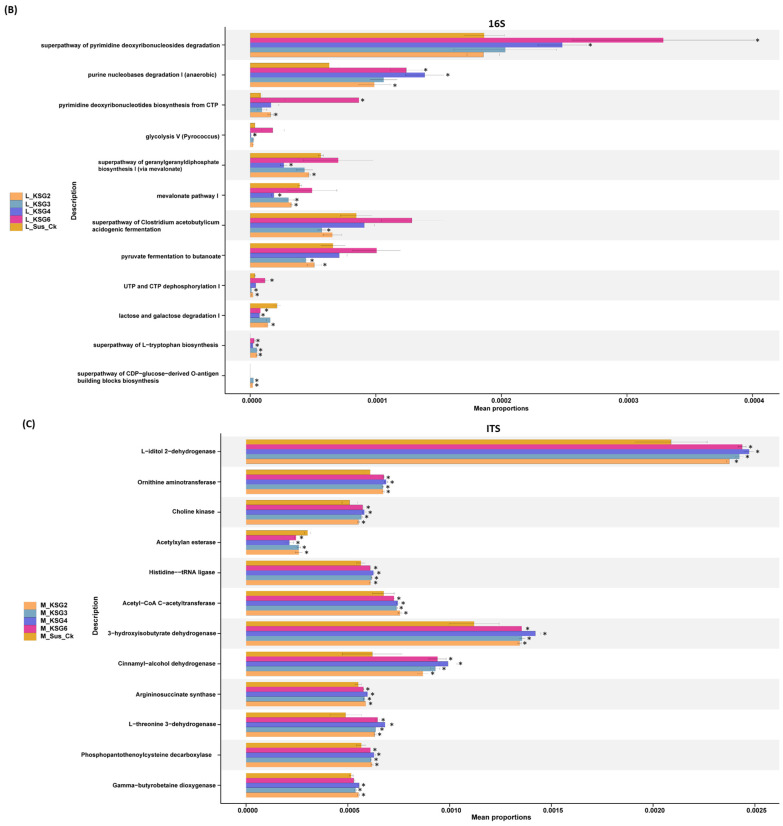
KEGG modules were significantly different (*p* < 0.05 *) between the resistant and susceptible sugar beet genotypes. (**A**) Mid (M) storage time point (bacteria; 16S); (**B**) late (L) storage time point (bacteria; 16S); and (**C**) mid (M) storage time point (fungi; ITS). Data are mean ± standard error of 4 replicates (each replicate consists of samples obtained from two roots). Sus_Ck: susceptible genotype; KSG2, KSG3, KSG4, and KSG6: resistant genotypes.

At the M storage time point, the majority of the detected bacterial microbiome-associated KEGG pathways were primarily down-regulated (*p* < 0.05) in the R lines, except for KSG2. A few examples of KEGG pathways that were highly down-regulated in the R lines at the M time point include the super pathways of pyrimidine deoxyribonucleoside degradation, the super pathway of thiamin diphosphate biosynthesis I, the super pathway of thiamin diphosphate biosynthesis II, etc. (Figure 4A). At the L time point, pathways that were primarily up-regulated in the R lines included purine nucleobase degradation I (anaerobic) and the super pathway of L-tryptophan biosynthesis (Figure 4B).

Pathway enrichment of the fungal microbiome could be resolved only at the M time point. The majority of the detected KEGG pathways were up-regulated in the R lines. A few examples of KEGG pathways that were highly up-regulated in the R lines include L-iditol 2-dehydrogenase, 3-hydroxyisobutyrate dehydrogenase, cinnamyl-alcohol dehydrogenase, etc. (Figure 4C).

### 2.4. Correlation Between Bacterial and Fungal Communities Across Genotypes

**Figure 5:**
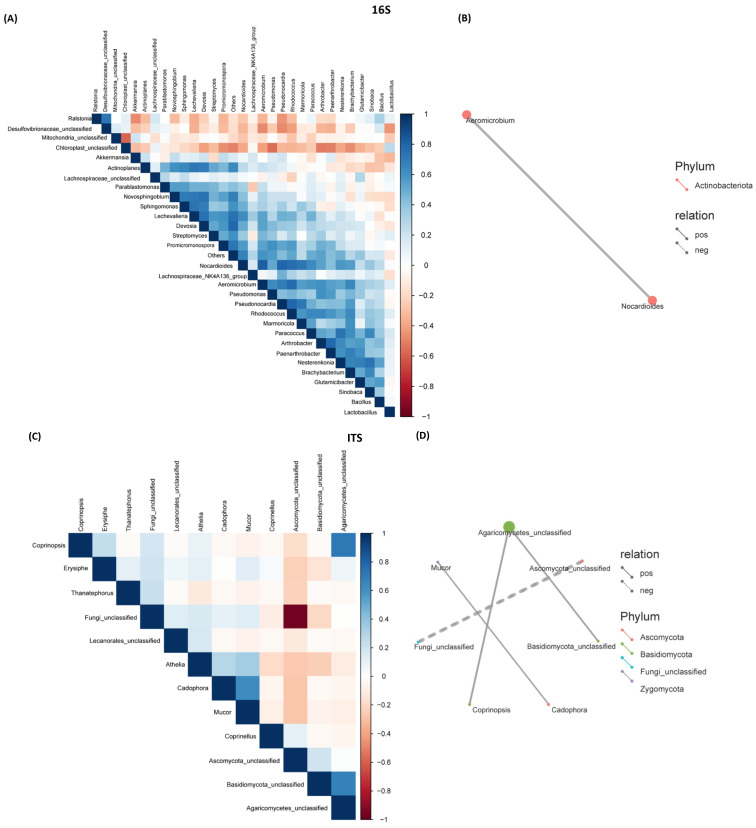
Sparse Correlations for Compositional data (SparCC). (**A**) Correlation heatmap between bacterial communities; (**B**) correlation network between bacterial communities; (**C**) correlation heatmap between fungal communities; and (**D**) correlation network between fungal communities. A solid line between two bacterial/fungal communities indicates a positive correlation and a dotted line indicates a negative correlation between them. The thicker the solid line, the higher the value of the positive correlation between them.

To understand the relationships between bacterial and fungal communities and if their co-occurrence was associated with post-harvest resistance and/or susceptibility, a correlation matrix (Figure 5A,C) and correlation networks (Figure 5B,D) were constructed, including all genotypes collected from both M and L time points. A strong positive correlation (+0.83) was observed between *Aeromicrobium* and *Nocardioides*.

In the case of the fungal microbiome, a moderate positive correlation (+~0.70) was observed between *Agaricomycetes* and *Basidiomycota*, and between *Agaricomycetes* and *Coprinopsis*. A moderate correlation (+0.62) was also observed between *Mucor* and *Cadophora*. A strong negative correlation (−0.96) was observed between *Ascomycota* and an unclassified fungal group.

### 2.5. Untargeted Metabolome Analysis of the Roots of Susceptible (S) and Resistant (R) Lines

**Figure 6:**
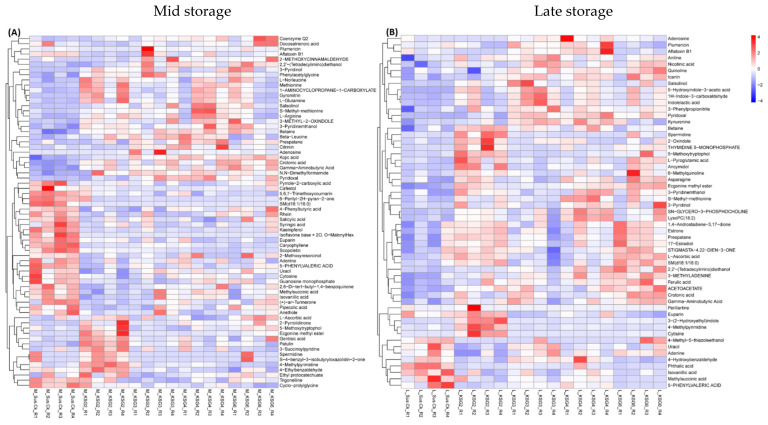
Untargeted metabolome analysis of sugar beet roots showed distinct differences between the resistant (R) and susceptible (S) lines at mid and late storage time points. Metabolites showing major differences between the R vs. S lines at the (**A**) mid storage stage and (**B**) late storage stage. Sus.Ck: susceptible genotype; KSG2, KSG3, KSG4, and KSG6: relatively resistant genotypes; M: mid and L: late storage time points.

Untargeted metabolome analysis was performed to understand changes in metabolites in the roots of the resistant and susceptible lines during prolonged indoor storage. Figure S5 shows the PCA analysis and clustering of all samples originating from the M and L time points. Overall, the genotypes maintained specific clustering patterns. Selected metabolites that showed the most changes between the R and S lines are presented in Figure 6. Metabolites that were significantly higher in the R lines, primarily in KSG3, KSG4, and KSG6 (vs. S), at the M time point (Figure 6A) include kojic acid, gamma aminobutyric acid (GABA), N,N-dimethylformamide, pyridoxal, etc. Examples of metabolites that were primarily higher in the R line, KSG2 (vs. all other lines), include 5-methoxytryptophol, ecgonine, spermidine (Spd), 4-methylpyrimidine, 4-ethylbenzaldehyde, etc. A few examples of metabolites higher only in the S line include euparin, caryophyllene, scopoletin, etc.

At the L time point (Figure 6B), the relative abundances of metabolites in the R lines (vs. S) exhibited a different pattern. Metabolites highly abundant in the R line, KSG2, included betaine, Spd, 5-methoxytrytophol, 3-(2-hydroxyethyl)indole, 4-methylpyrimidine, cytisine, etc. Metabolites that were predominantly higher in the R lines (vs. S) were asparagine, ecgonine methyl ester, crotonic acid, GABA, 5-hydroxyindole-3-acetic acid, indoleacetic acid, etc. Metabolites that were only higher in the S line included phthalic acid, isovanillic acid, methylsuccinic acid, etc.

**Figure 7:**
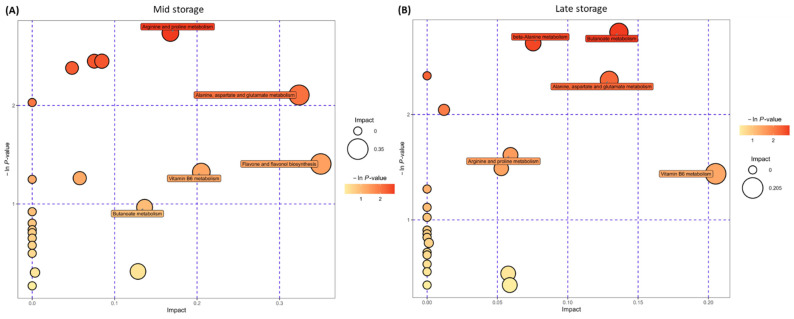
Pathway enrichment analysis of sugar beet roots. (**A**) Mid storage time point and (**B**) late storage time point. Data are mean ± standard error of 4 replicates (each replicate consists of samples obtained from two roots).

Pathway enrichment analysis showed a higher enrichment of metabolites primarily associated with arginine, proline, alanine, aspartate, and glutamate metabolism, followed by flavone and flavonoid metabolism in the R lines at the M stage (Figure 7A). At the L stage, similar amino acids, in addition to butonate metabolism pathways, were enriched in the R lines (Figure 7B).

### 2.6. Carbohydrate Content in the Roots of Susceptible (S) and Resistant (R) Lines

**Figure 8:**
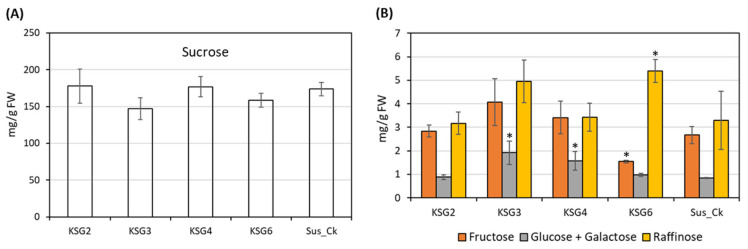
Carbohydrate content in the roots showed small changes between the resistant and susceptible lines at the late storage time point. Cellular contents (mg/g FW) of: (**A**) sucrose and (**B**) fructose, glucose and galactose, and raffinose. Data are mean ± standard error of 4 replicates (each replicate consists of samples obtained from two roots); *p* < 0.05 * between the susceptible (Sus_Ck) and resistant genotypes (KSG2, KSG3, KSG4, and KSG6).

The root carbohydrate content in the R and S lines at the late storage stage was also analyzed. The sucrose content in the root samples ranged between 147–178 mg/g fresh weight (FW) (Figure 8A). No significant differences in sucrose content were noticed between the R and S lines after post-harvest storage. The concentrations of other sugars in the sugar beet roots detected in this study were between 0.84–5.40 mg/g FW (Figure 8B). In general, no significant difference in fructose content was observed between the R and S lines, except for the KSG6 line, which showed a 42% lower fructose content than the Sus_Ck line. Glucose and galactose contents were 28–52% higher in the KSG3 and KSG4 lines, and raffinose content was 64% higher in KSG6 (vs. Sus_Ck).

### 2.7. Correlation Analysis Between the Root Microbiome and Metabolome

**Figure 9:**
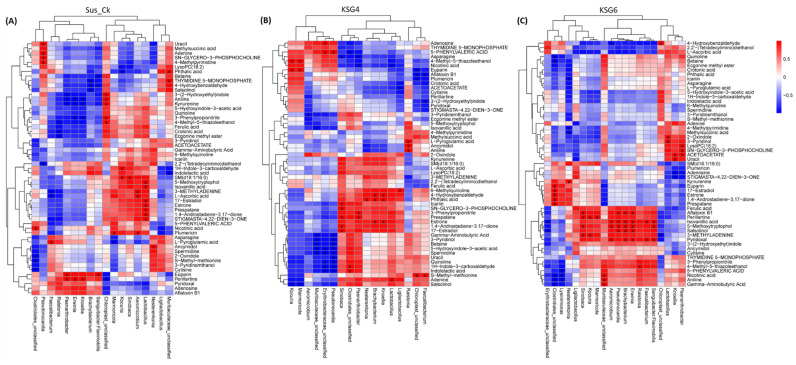
Correlation analysis between the root microbiome and metabolome at the late storage time point reveals a distinct pattern in the two highly resistant genotypes vs. the susceptible genotype. (**A**) Susceptible genotype (Sus_Ck); (**B**) resistant genotype, KSG4; and (**C**) resistant genotype, KSG6. Data are mean ± standard error of 4 replicates (each replicate consists of samples obtained from two roots). An ‘X’ sign inside the rectangular boxes in the heatmap indicates *p* < 0.05.

As the microbial composition in sugar beet roots is critical for root resistance against pathogens, we therefore performed a correlation analysis to understand the relationships between the root microbiome and root metabolites. The bacterial genera in either the R or S lines that were significantly associated with specific root metabolites were identified (Figure 9). Two of the highly resistant R lines (KSG6 and KSG4), along with the S line, Sus_Ck, are presented in Figure 9, and other R lines (KSG2 and KSG3) are presented in Figure S6. In Sus_Ck (Figure 9A), some examples of significant positive correlations between bacteria (putatively associated with susceptibility) and metabolites include *Lactobacillus*, which is positively correlated with metabolites such as 17-estradiol, estrone, prespatane, etc.; *Ralstonia*, which is negatively correlated with metabolites such as crotonic acid, ecgonine methyl ester, etc.; and *Marmoricola*, which is positively correlated with plumericin and negatively correlated with L-pyroglutamic acid. Examples of some significant positive correlations between bacteria (putatively associated with resistance) and metabolites in the R line KSG6 (Figure 9C) include *Nesterenkonia*, which is positively correlated with kynurenine, and *Paenarthrobacter*, which is positively correlated with metabolites including LysoPC, sn-glycero-3-phosphocholine, and acetoacetate. In KSG4 (Figure 9B), *Erythrobacteraceae* is positively correlated with adenosine and 5-phenylvaleric acid. In KSG2 (Figure S6A), *Muribaculaceae* is positively correlated with crotonic acid, methylsuccinic acid, etc., *Erythrobacteraceae* is positively correlated with GABA and 3-pyridinol, *Paenarthrobacter* is positively correlated with kynurenine and 3-methyladenine, and *Brachybacterium* is positively correlated with 4-hydroxybenzaldehyde, 3-methyladenine, and kynurenine. Some of these putative resistance-associated bacteria showed positive/negative correlations with other metabolites in KSG3 (Figure S6B).

Analysis of the fungal microbiome in the roots of R and S lines identified fungal genera predominantly associated with pathogenicity (Figure S7). We similarly performed a correlation analysis between the fungal genera and root metabolites. In Sus_Ck (Figure S7A), examples of significant positive correlations between fungi and metabolites included *Athelia* and *Cadophora*, which were positively correlated with perillartine, and *Mucor* with adenosine. Examples of negative correlations included *Mucor* with quinoline and 3-phenylpropionitrile, as well as *Athelia* and *Cadophora* with kynurenine, aniline, etc. Examples of negative correlations in the R line KSG2 (Figure S7B) included *Athelia*, which was negatively correlated with LysoPC and sn-glycero-3-phosphocholine, and *Lecanorales*, which was negatively correlated with Spd and quinoline. In KSG3 (Figure S7C), *Athelia* was positively correlated with plumericin and *Erysiphe* was negatively correlated with L-ascorbic acid, 5-phenylvaleric acid, prespatane, etc. In KSG4 (Figure S7D), *Athelia* was positively correlated with plumericin. Negative correlations were observed between *Athelia* and *s*-methylmethionine, as well as *Coprinellus* and L-pyroglutamic acid. In KSG6 (Figure S7E), *Athelia* was positively correlated with 17-estradiol, and *Erysiphe* was positively correlated with pyridoxal, isovanillic acid, etc. Examples of negative correlations in KSG6 included *Athelia*, which was negatively correlated with crotonic acid and GABA.

### 2.8. Resistant Lines Exhibited Lower Disease Symptoms vs. the Susceptible Line

**Figure 10:**
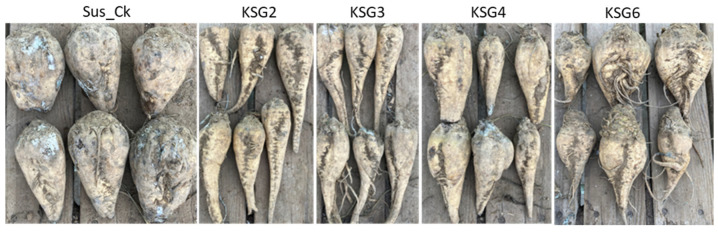
Disease symptoms on sugar beet roots at the late storage time point under indoor storage conditions. Representative samples showing surface coverage with fungal growth in the susceptible genotype (Sus_Ck) and resistant genotypes (KSG2, KSG3, KSG4, and KSG6).

The R lines showed significantly lower (2–5%) root surface area coverage with disease symptoms in comparison to the Sus_Ck line, which exhibited 18% coverage of root surface at the L time point (Figure 10; Table S6).

## 3. Discussion

The loss of sucrose during the post-harvest storage of sugar beets is due to root respiration and can be increased by diseases [1,2,3,4,30,32,38]. Host plant genotype-specific factors such as the microbiome and metabolome can also play a critical role in improving the storability of sugar beet roots [29,30,31,39]. The plant microbiome plays a key role in mitigating biotic and abiotic stresses and improving overall plant growth and fitness [40]. Therefore, identification of markers related to beneficial microbes and/or metabolites will be key in selecting sugar beet cultivars exhibiting higher resistance to storage pathogens pertinent to specific geographical areas to minimize sucrose loss. The current study was undertaken to understand the association between the root microbiome and metabolites in the context of greater resistance to post-harvest pathogens and better storability.

### 3.1. Microbial Diversity in the Resistant Lines and Their Putative Roles in Post-Harvest Disease Resistance

The R and S lines showed temporal differences in bacterial phyla and genera, indicating their putative roles during their prolonged storage time (~5 months) following harvest. Higher relative abundances of the bacterial phyla *Firmicutes* and *Desulfobacterota*, especially in the S line at the M time point (Figure 1 and Figure S2A), were observed. These observations are in line with earlier findings in sugar crops. Abiotic stresses such as waterlogged conditions in sugarcane facilitated an increase in the populations of anaerobes belonging to the bacterial phyla *Firmicutes* and *Desulfobacterota*. The phylum *Firmicutes* includes Gram-positive bacteria such as *Leuconostoc*, *Clostridium*, *Lactobacillus*, and *Weissella*, which can cause sucrose loss in sugar beet and sugarcane [41,42,43]. A higher abundance of *Firmicutes*, predominantly represented by the order *Lactobacillales*, was observed in decaying sugar beet root samples [42]. The results presented here, along with earlier findings, indicate that specific members belonging to *Firmicutes* contribute to susceptibility in stored sugar beet roots, adversely affecting their storability. A higher relative abundance of *Desulfobacterota* in the R lines, KSG4 and KSG6, was observed at the L time point (Figure S2B). Higher disease resistance in KSG4 and KSG6, and the known function of members belonging to this phylum in resistance against pathogens, might indicate their role in improving the resistance of sugar beet root against storage pathogens. Future characterization of specific members of *Desulfobacterota* will determine their precise role in the post-harvest storage of sugar beet roots. Another bacterial phylum, *Patescibacteria*, known for antagonistic effects against fungal pathogens, has been isolated from diverse environments including plants and soils [44,45]. Their role in host plant resistance against pathogens varies depending upon plant species, pathogens involved, and environmental conditions. *Patescibacteria*, as a component of the plant microbiome, can interact with other microbes, thereby affecting their populations and positively affecting plant defense responses. In a more recent study, a higher abundance of *Saccharimonadales* (members of *Patescibacteria*) was observed in a sugar beet cultivar resistant to root rot [45]. A higher abundance of *Patescibacteria* in the R lines at the M time point (Figure S2) is in line with earlier findings indicating their putative roles in activating early resistance responses against sugar beet storage pathogens. Although no major differences in alpha and beta diversity among bacteria and fungi were observed between the R and S lines, specific members of the microbial communities may play key roles in disease resistance. Biomarker analysis revealed the presence of bacterial taxa putatively associated with resistance against fungal pathogens, including *Micrococcaceae*, *Nesterenkonia*, *Paenarthrobacter*, *Brachybacterium*, *Micrococcales*, *Bacilli*, etc., and these were highly abundant in the most resistant line, KSG6, at both M and L time points (Figure 3), indicating their role in resistance during a prolonged storage period. A highly positive correlation between *Aeromicrobium* and *Nocardioides* (Figure 5B), bacteria genera with known functions in resisting fungal/bacterial pathogens [46,47], as well as a higher abundance of *Aeromicrobium* in the R line KSG6, indicates their co-occurrence and contribution towards resistance against sugar beet storage pathogens in a genotype-specific manner. The bacterial genera putatively associated with resistance identified in this study somehow vary among the R lines, as reported in some earlier studies. In sugar beet clamps collected from Austria and Germany, bacterial genera highly represented in healthy roots included *Flavobacterium*, *Pseudarthrobacter*, and *Pseudomonas*, whereas decaying roots showed higher abundances of *Lactobacillus*, *Gluconobacter*, and *Leuconostoc* [29,31]. The presence of *Lactobacillus*, *Leuconostoc*, and *Gluconobacter* was also reported in decaying sugar beets collected from southern Idaho and southeastern Oregon in the U.S. [42]. This suggests that a wide range of beneficial bacteria might play roles against post-harvest storage pathogens and contribute to resistance in a sugar beet genotype-specific manner. The mechanisms by which the microbiome affects plant disease resistance include the production of metabolites that interact with other beneficial microbes and reconstitute microbiome composition, modulate host defense pathways, and/or possess antimicrobial activities [40]. Higher enrichment of the bacterial community associated with metabolic pathways, such as the super pathway of L-tryptophan biosynthesis, was observed in the R lines at the L time point when disease symptoms were at their maximum (Figure 4B). A higher abundance of Trp-derived SMs (e.g., quinoline) in the R lines (Figure 6) and the known roles of Trp/Trp-derived metabolites (discussed later) are indicative of the microbial contribution towards overall resistance against sugar beet storage pathogens.

The S line showed a higher abundance of fungal taxa, namely *Mucor*, *Athelia*, *Ascomycota-related*, *Cadophora*, *Basidiomycota-related*, etc. (Figure 1D), which are known to produce disease symptoms in sugar beet roots during post-harvest storage. It should be noted that the storage temperatures recorded during the last few months of storage were below freezing (Figure S5A) in this study. The prevalence of fungal pathogens reported in sugar beet piles varies depending upon environmental parameters during storage seasons, geographical area, outdoor/indoor storage, genotypes, etc. An earlier study investigating the role of temperature on sugar beet post-harvest fungal pathogens during storage revealed the prevalence of *Botrytis cinerea* at 8 °C, whereas members belonging to the fungal genera *Fusarium* and *Penicillium* were dominant at 20 °C [30]. Several post-harvest pathogens, including *Botrytis cinerea*, *Athelia*-like sp., *Fusarium* spp., *Penicillium* spp., and *Mucor* spp., have been reported in sugar beet growing areas in the U.S. [4,6]. Additional opportunistic microbial communities associated with storage diseases were also reported in post-harvest sugar beet roots as rotted lesions serve as entry points for the internal root tissues [42]. Fungal genera associated with decaying sugar beets collected from clamps in Austria and Germany were predominantly represented by *Candida* and *Penicillium* [31]. Fungal biomarkers identified in the rhizosphere and outer endosphere of harvested beets associated with better storability were *Plectosphaerella* and *Vishniacozyma*. Although a strong negative correlation (−0.96) was observed between Ascomycota and an unclassified fungal group, we were unable to resolve it at the genus level (Figure 5D).

### 3.2. Metabolic Signatures of Resistant Lines Contributing to Resistance

An untargeted metabolomics analysis of sugar beet roots showed significant changes in metabolites between the R and S lines that varied with time and the intensity of disease symptoms. Examples of metabolites that showed a higher relative abundance in the R lines (vs. S) at the M time point (Figure 6A) included gamma-aminobutyric acid (GABA), Gln, spermidine (Spd), pyridoxal 5-methoxytryptophol, ecgonine, kojic acid, etc. Some of the metabolites that were higher in the R lines at the L time point and overlapped with M included GABA, Gln/Gln-derivative, Spd, crotonic acid, etc. Other metabolites, such as betaine, 5-methoxytrytophol, 3-(2-hydroxyethyl)indole, 4-methylpyrimidine, asparagine (Asn), crotonic acid, pyroglutamic acid (PGA), etc., were higher in the R lines at the L time point (Figure 6B). Pathway enrichment analysis revealed differentially regulated metabolites predominantly associated with Arg and Pro metabolism, followed by Ala, Asp, and Glu metabolism, during the M storage time point (Figure 7A). A shift in metabolism during the L storage time point associated with beta-alanine and butanoate metabolism (Figure 7B) indicated the temporal regulation of metabolism in the roots and the possible involvement of beta-alanine/butanoate metabolism-associated metabolites, such as 4-aminobutanoate, acetoacetate, Spd, and uracil, in resistance in the R lines (vs. S), which exhibited few disease symptoms (Figure 10).

Amino acids (AAs) in plants play critical roles in the synthesis of proteins and secondary metabolites (SMs) associated with growth and tolerance to biotic/abiotic stresses [48,49]. Besides serving as reserves for nitrogen (N) and (C), free AAs also act as osmoregulants in plants [50]. This is especially true in the context of water loss by sugar beet roots during post-harvest storage, which could positively affect root respiration and loss of sucrose [32]. Higher abundances of Gln/Gln-derivative, GABA, Spd (triamine), and other related AAs were observed in the R lines at the M and L time points (Figure 6B) when the disease pressure was at its maximum (Figure 10). A higher amount of Gln in sugar beet genotypes showing greater resistance to post-harvest pathogens [39] and the data presented here indicate a protective role of these metabolites against post-harvest pathogens. Glutamine represents a major portion of the alpha-amino N in sugar beet roots and acts as a major substrate to produce the majority of the amino acids in plants [39,51]. The role of Gln in plant disease resistance has been shown partly through its interaction with the salicylic acid pathway, triggering plant immunity [51,52]. Besides Glu/Gln, Arg also serves as a reservoir of N in the roots, feeding the polyamine (PA) biosynthetic pathway and producing the non-protein amino acid (AA), GABA, from the diamine putrescine. Gamma-aminobutyric acid is also synthesized directly from glutamate (Glu) through glutamate decarboxylase [53] and can directly enter the tricarboxylic acid (TCA) cycle, contributing to energy production. The role of GABA linking to the TCA cycle might be highly relevant to the current context due to continued root respiration and the production of other SMs to combat pathogens during prolonged storage conditions. Gamma-aminobutyric acid has been shown to confer biotic/abiotic stress tolerance in plants through its antioxidant properties and interaction with other signaling molecules such as Ca^2+^, H_2_O_2_, polyamines (PAs), salicylic acid, nitric oxide, etc. [54,55]. The protective role of the PA and Spd against pathogens from the host plant’s perspective depends upon the direct regulation of defense-related genes, as Spd conjugates possess antimicrobial properties and the catabolism of Spd produces H_2_O_2_, which is involved in defense signaling [56,57]. Some of our observations on higher amounts of specific AAs in the roots of sugar beet genotypes used in this study and the higher resistance to storage and fungal pathogens overlap with earlier findings in sugar beet [39]. The relationship between aromatic amino acids (Trp, Tyr, and Phe) and greater resistance to post-harvest diseases in sugar beet genotypes has been reported earlier [39]. Ferulic acid, a compound derived from Phe through phenylpropanoid metabolism, was higher in the R lines (vs. S) at the L time point (Figure 6B). Ferulic acid has been demonstrated to improve the post-harvest storage quality of crops through wound healing, thereby improving cell wall rigidity, inhibiting pathogens (antimicrobial properties), reducing water loss, and increasing antioxidant potential [58,59,60]. Similarly, the alkaloid quinoline (derived from Trp), known to possess both antifungal and antibacterial properties [61], was higher in the R lines (vs. S) at this time point. The data presented here further delineate putative mechanisms through which aromatic AAs might confer resistance against post-harvest pathogens. This might also suggest common strategies and sugar beet genotype-specific biochemical traits associated with the better storability of roots. However, free AAs are considered impurities in sugar beet roots, since they negatively affect sucrose purification during industrial processing [62]; however, there may be a threshold for specific AAs that are essential for disease resistance during storage. Taken into consideration, the data presented from this study and earlier reports indicate the critical roles of AAs involved in the production of specialized N-containing SMs contributing to resistance against pathogens and other abiotic stresses during post-harvest storage.

Among other compounds, PGA, an organic acid, showed a higher abundance in the R lines KSG2 and KSG6 (vs. S) at the L storage time point (Figure 6B). Pyroglutamic acid is produced either from Glu or the degradation of glutathione [63]. Besides its direct antifungal and antibacterial properties [64], PGA has also been shown to activate plant defense pathways associated with improving antioxidant reserves [65]. Considering the higher abundance of PGA in the R lines in this study, as well as an earlier report in sugar beets [39], PGA may have a potential role against post-harvest pathogens. Several other compounds, such as betaine, prespatane, S-methylmethionine, etc., which were higher in the R lines, are not fully understood with regards to their role in disease resistance in sugar beets. Therefore, further studies will be required to understand their precise contributions towards resistance against fungal pathogens.

Global metabolite analysis (Figure 6) of the roots of R and S lines primarily represented metabolites originating from the plant. Distinguishing metabolites that are exclusively of microbial origin vs. plants is challenging as a majority of these metabolites are common to both plants and microbes. Examples of metabolites that were exclusively of microbial origin include citrinin, gyromitrin, kojic acid, ecgonine methyl ester, aflatoxin, patulin, etc. Some of these metabolites have been shown to have beneficial roles against pathogens. The alkaloid ecgonine methyl ester has been shown to be produced by *Bacillus subtilis* [66]. The role of this alkaloid in biotic stress alleviation in plants, its higher abundance, and the higher relative abundance of *Bacillus* sp. in the R lines might indicate its putative role in resistance against fungal pathogens. The mycotoxins, aflatoxin and patulin, which were detected in some samples, are known to be produced by *Aspergillus flavus* and *Penicillium* spp., respectively. Interestingly, both pathogens were either undetected or insignificant in the R and S lines at M and L time points. Whether these mycotoxins can be produced by other microbes is unknown. No direct meaningful correlations could be obtained from the correlation analysis of these mycotoxins and related fungal genera; therefore, these relationships could not be resolved.

### 3.3. Correlation Between Root Metabolites and the Microbiome Indicates the Role of Host Genotype in Resistance

The correlation between sugar beet root metabolites and root bacteria known for their roles in resistance/susceptibility provided greater insights on the regulation of the microbiome by host genotype. Some specific examples include a positive correlation between *Lactobacillus* (susceptibility related) and metabolites such as 17-estradiol, estrone, prespatane, etc. On the other hand, *Ralstonia* (susceptibility related) was negatively correlated with metabolites such as crotonic acid, ecgonine methyl ester, etc. Examples of positive correlations between bacteria (resistance related) and metabolites in the R lines (Figure 9 and Figure S6) included *Nesterenkonia* with kynurenine (in KSG6) and methylsuccinic acid (in KSG3 and KSG6), and *Nesterenkonia* with icariin (in KSG2 and KSG6). *Paenarthrobacter*, putatively associated with resistance, was positively correlated with metabolites including LysoPC, sn-glycero-3-phosphocholine, and acetoacetate (in KSG6, Figure 9C). In KSG4 (Figure 9B), *Erythrobacteraceae* was positively correlated with adenosine and 5-phenylvaleric acid. Kynurenine (KYN), produced from Trp, serves as a direct precursor for kynurenic acid, anthranilic acid, 3-hydroxykynurenine (3 − HK), and other downstream metabolites such as quinoline [66]. The role of KYN in plants has been demonstrated to be as an auxin suppressant, negatively affecting root growth. Although the relationship between KYN and the gut microbiome in human health has been demonstrated [67], KYN’s effect on the microbiome composition in plants and any role in sugar beet disease resistance has not been investigated. Similarly, icariin, an isopentenyl flavonoid, has been demonstrated to increase beneficial bacteria in mammalian guts [68]. A higher abundance of metabolites such as KYN and icariin in the R lines at the L time point, along with a higher abundance of beneficial bacteria (disease resistance) and greater resistance against fungal pathogens in the R lines, might highlight their putative roles in resistance. Associations between other compounds, such crotonic acid, methylsuccinic acid, LysoPC, and sn-glycero-3-phosphocholine, and their bacterial counterparts are not fully understood at this moment. Though there are overlaps between specific plant metabolites and resistance-associated bacteria in specific R lines, there are instances where the resistance-related bacteria correlated with different metabolites in the other R lines and vice versa. Correlation analysis between root metabolites and the fungal microbiome identified key root metabolites putatively associated with resistance against fungal pathogens in the R lines (Figure S7). Several metabolites such as s-methylmethionine, LysoPC, sn-glycero-3-phosphocholine, crotonic acid, and GABA identified in the R lines showed a negative correlation with *Athelia*. Other fungal pathogens such as *Erysiphe* showed a negative correlation with L-ascorbic acid, 5-phenylvaleric acid, prespatane, etc., as well as *Coprinellus* with L-pyroglutamic acid. Some of the resistance-related metabolites, such as LysoPC, sn-glycero-3-phosphocholine, crotonic acid, and GABA, overlapped with the bacteriome data, indicating possible mechanisms through which these metabolites may control fungal pathogens. Overall, these data suggest that the root biochemical environment may control the relative abundance of beneficial bacterial species in a genotype-specific manner. The data presented here, along with earlier findings, shed light on the plant metabolites that are associated with a higher relative abundance of specific beneficial bacteria and plant disease resistance [69]. Overall, this study provides insights into how the dynamics of microbiomes and metabolites can contribute to improving the storability of sugar beets. Future studies targeting the functional roles of key microbiomes and metabolites, as well as specific responses to distinct components, will warrant an understanding of underlying mechanisms important for retaining the post-harvest health of sugar beets.

## 4. Materials and Methods

### 4.1. Storage Conditions of Sugar Beet Roots, Evaluation of Roots, and Sample Collection During Prolonged Indoor Storage

Sugar beet roots of resistant [mutational breeding lines: KSG2 (KEMS06), KSG3 (KEMS08), KSG4 (KEMS08-600); genetic selection from Polish background: KSG6 (KPS25)] and susceptible [Sus_Ck; commercial variety] lines to post-harvest storage-related diseases were stored in an indoor commercial sugar beet storage facility at Paul, ID (USA) [34,35,36]. The indoor post-harvest storage temperature and relative humidity from mid-Oct to mid-March are presented in Figure S8. Sugar beet roots were weighed, evaluated for disease symptoms (percent surface coverage of roots with pathogen growth), and root samples were collected from both R and S lines at M (end of December; ~3 months after harvest) and L storage (mid-March; ~5 months after harvest) time points for microbiome and metabolite analyses. Root samples from each genotype were collected in 4 replicates at the M and L time points and each replicate consisted of samples collected from two sugar beet roots. Root samples (10 roots/genotype) were evaluated at both M and L time points for percent visual surface coverage of roots with disease symptoms out of total root surface area. Following sample collections, root tissues (fresh weight) were flash-frozen in liquid N and stored at −80 °C until further processing for microbiome and metabolome analyses. Root samples were pulverized under ultra-low temperatures using Geno/Grinder 2010 (SPEX SamplePrep, Metuchen, NJ, USA) before extraction of genomic DNA (gDNA) and metabolites.

### 4.2. Genomic DNA Extraction, PCR Amplification, and 16S rRNA and ITS Sequencing

Genomic DNA (gDNA) from sugar beet root samples collected at mid and late storage stages were extracted using the Plant/Fungi DNA Isolation Kit (Norgen Biotek Corp., Thorold, ON, Canada) according to the manufacturer’s protocol and stored at −20 °C until further use. The extracted DNA was used for PCR amplification of the V3–V4 hypervariable region using the 338F_5′-ACTCCTACGGGAGGCAGCAG-3′ and 806R_5′-GGACTACHVGGGTWTCTAAT-3′ primer pairs for 16S rRNA amplification [70]. The ITS2 region of the fungal rRNA gene’s small subunit was amplified using the primers ITS7: F_5′-GTGARTCATCGAATCTTTG-3′ and ITS4: R_5′-TCCTCCGCTTATTGATATGC-3′ [71]. The 5′ ends of the primers were tagged with sample-specific indexing barcodes and universal adapters. Polymerase chain reaction (PCR) was performed in a total reaction volume of 25 μL containing 25 ng of template DNA per reaction. The PCR conditions included: initial denaturation at 98 °C for 30 s; 32 cycles of denaturation at 98 °C for 10 s (35 cycles for ITS amplification), annealing at 54 °C for 30 s, and extension at 72 °C for 45 s; followed by a final extension step at 72 °C for 10 min. The PCR products were confirmed through 2% agarose gel electrophoresis. The PCR products were then purified using AMPure XT beads (Beckman Coulter Genomics, Danvers, MA, USA) and quantified using a Qubit (Invitrogen, Waltham, MA, USA) system. The amplicon pools were prepared for sequencing. The size and quantity of the amplicon libraries were measured using an Agilent 2100 Bioanalyzer (Agilent, Santa Clara, CA, USA) and a Library Quantification Kit for Illumina (Kapa Biosciences, Woburn, MA, USA), respectively. The libraries were sequenced on a NovaSeq PE250 platform with paired-end reads (2 × 250 bp) according to the manufacturer’s protocols (LC Sciences, Houston, TX, USA).

### 4.3. Data Analysis

The metagenomic reads obtained from the 16S sequencing were processed using the microbiome data analysis pipeline from LC Sciences (Houston, TX, USA). The workflow included: (1) assessment of read qualities by removing short sequences (<150 bp) and ambiguous base calls using a maximum expected error threshold of 1.0, (2) classifying unique sequences after removing sequencing/PCR errors and chimera sequences, (3) dereplication, through which all identical sequences were combined into unique sequence reads, and (4) assignment of a zero-radius operational taxonomic unit (zOTU). Taxonomic classification of final zOTUs were performed using BLASTn against the NCBI (www.ncbi.nlm.nih.gov) database.

Paired-end reads generated from sequencing were assigned to specific samples based on unique barcodes, followed by removing the barcode and primer sequences. The reads were then merged using FLASH. Specific filtering conditions were used on raw reads to obtain high-quality clean tags using fqtrim software (v0.94). Chimeric sequences were filtered using Vsearch software (v2.3.4). Dereplication was performed using DADA2, resulting in feature sequences being obtained. Sequences exhibiting ≥97% similarity were assigned to the same OTUs. Representative sequences were chosen for each OTU. Alpha diversity and beta diversity were calculated by normalizing to the same sequences randomly. Through the SILVA (release 132) classifier, feature abundance was normalized using relative abundance for each sample. To measure the complexity of species diversity in each sample, alpha diversity was applied using indices, observed_OTUs, and Shannon. These indices were measured using QIIME2. The zOTU abundance tables were used to estimate beta diversity using principal coordinate analysis (PCoA). This helped to examine differences among samples regarding species complexity. Cluster analysis was performed using QIIME2 (v1.8.0). The graphs were constructed using the R package (v3.5.2). Blast was used for sequence alignment, and the feature sequences were annotated using the SILVA database (Release 138; 2019) for each representative sequence.

To identify differentially abundant families among treatments for potential biomarker/s [72], linear discriminant analysis effect size (LEfSe) was used. The relative abundance of taxonomic features obtained through the greengenes 13_8 database was then used as input for the analysis of LEfSe using the huttenhower server (https://huttenhower.sph.harvard.edu/galaxy/, accessed on 20 April 2023). The default LDA effect size alpha value (*p* = 0.05) and LDA score (2.0) settings were used to identify significant differences among groups. The Phylogenetic Investigation of Communities by Reconstruction of Unobserved States (PICRUSt) algorithm [73] was used for functional assignment of microbial communities. The greengenes 13_8 database was used to cluster closed reference OTUs and the resulting ‘.biom’ files were used as inputs for PICRUSt (http://huttenhower.sph.harvard.edu/galaxy/, accessed on 20 April 2023). After normalization of the input OTU table, functional predictions of the metagenome were performed using the Kyoto Encyclopedia of Genes and Genomes (KEGG) database [74,75] as a reference. Co-occurrence between bacterial communities was determined using the Sparse Correlations for Compositional data (SparCC) rank correlation coefficients (Python 2.6.1). From the original dataset, a random simulation of 100 datasets was created. Pseudo-*p*-values were calculated by estimating the number of the dataset that produced the same correlation as the real data [76].

The Illumina MiSeq platform was used for ITS sequencing according to the manufacturer’s recommendations (LC-Bio). Paired-end reads were assigned to the samples based on their unique barcodes. The raw reads were processed by removing the barcodes and primer sequences. Paired-end reads were merged using PEAR (v1.2.8). Quality filtering of the raw tags was performed using specific filtering conditions to obtain high-quality clean tags according to fqtrim (version 0.94). Chimeric sequences were filtered using Vsearch (v2.3.4). Sequences with ≥97% similarity were assigned to the same operational taxonomic units (OTUs) using Vsearch (version 2.3.4). Representative sequences were chosen for each OTU following the assignment of taxonomic data to each representative sequence using the RDP (Ribosomal Database Project; 2019 version) classifier. The differences between dominant species in different groups and multiple sequence alignments were conducted using the mafft software (version 7.310) to obtain phylogenetic relationships between different OTUs. The abundance information of OTUs was normalized using a standard sequence number corresponding to the sample with the least sequences. Alpha diversity analysis to analyze the complexity of species diversity for a given sample was performed using observed_OTUs and Shannon through QIIME (version 1.8.0). Beta diversity analysis to evaluate sample differences with regard to species complexity was calculated using Principal Coordinates Analysis (PCoA) and cluster analysis using QIIME software (version 1.8.0).

### 4.4. Untargeted Metabolome Analysis

Approximately 50 mg of finely ground sugar beet root tissues (fresh weight) were extracted in 800 μL of 80% methanol. The samples were vortexed, followed by grinding at 65 HZ for 180 s and sonicating at 4 °C for 30 min. This was followed by incubation at −40 °C for 1 h, vortexing for 30 s, and incubation at 4 °C for 0.5 h. The samples were then centrifuged at 12,000 rpm for 15 min at 4 °C. The supernatants were transferred to separate centrifuge tubes and incubated at −40 °C for 1 h. This was followed by centrifugation at 12,000 rpm for 15 min at 4 °C. A 200 μL volume of the supernatant from each sample was mixed with 5 μL of the internal standard (0.14 mg/mL 2-chlorophenyl alanine) and transferred to the injection vial.

Metabolites were analyzed using an LC-MS (Waters, UPLC; Thermo, Q Exactive) system with an attached Acquity UPLC HSS T3 (2.1 × 100 mm; 1.8 μm) chromatographic column. The chromatographic separation conditions were column temperature: 40 °C; flow rate: 0.300 mL/min; mobile phase composition A: water (0.05% formic acid), B: acetonitrile; injection volume: 5 μL; autosampler temperature: 4 °C. The mobile phase gradient elution program is presented in Table 2 below.

Mass spectrometry detection parameters for electrospray ionization (ESI; positive ion mode) included a heater temperature of 300 °C; sheath gas flow rate of 45 arb; auxiliary gas flow rate of 15 arb; sweep gas flow rate of 1arb; spray voltage of 3.0 KV; Capillary Temp of 350 °C; and S-Lens RF Level of 30%. The scan mode parameters were first-level full scan (Full Scan, *m*/*z* 70~1050) and data-dependent two-stage mass spectrometry scan (dd-MS2, TopN = 10). The resolutions were 70,000 (MS1) and 17,500 (MS2).

The raw data were converted to the mzXML format using ProteoWizard and processed using an in-house (Lifeasible; Shirley, NY, USA) program developed using R. XCMS was used for peak detection, extraction, alignment, and integration. An in-house MS2 database (LifeasibleDB) was applied for metabolite annotation. The cutoff for annotation was set at 0.3. The raw files (.raw) were then imported into Compound Discoverer3.1 (CD) software for spectral processing and a database search was carried out in order to obtain qualitative and quantitative results for the detected metabolites. This was followed by quality control to ensure the accuracy and reliability of the data. Multivariate analysis including principal component analysis (PCA) and partial least squares discriminant analysis (PLS-DA) was performed to detect any differences in metabolites among different groups. Hierarchical clustering (HCA) and metabolite correlation analysis revealed relationships between samples and metabolites. The biological significance of metabolites was identified through enrichment of metabolites with specific metabolic pathways. Metabolites were visualized using the ‘pheatmap’ package [77] in R (version 1.0.12) with default parameters except for scale = “row” (https://CRAN.R-project.org/package=pheatmap, accessed on 27 January 2024). Correlation analyses were performed using the cor.test function in R.

### 4.5. Carbohydrate Analysis

Carbohydrate extractions were performed according to the method described earlier [78]. Approximately 100 mg (fresh weight) of finely ground sugar beet root tissues (stored at −80 °C) were mixed with 1 mL of 80% absolute ethanol in 1.5 mL centrifuge tubes, followed by heating in a water bath at 65 °C for 30 min. The samples were then cooled for 5 min at room temperature, vortexed at medium speed on a multi-tube vortexer (VX-2500) for 2 min, and centrifuged at 13,000 rpm for 8 min. The supernatants were filtered using 0.45 um nylon syringe filters fitted in 1.5 mL microfuge tubes. The filtrates (extracts) were stored at −80 °C until analysis. Five soluble carbohydrates were analyzed using an AB Sciex 5600 triple time-of-flight mass spectrometer system (Framingham, MA, USA) according to the method described earlier [79]. A Waters (Milford, MA, USA) Acquity UPLC BEH Amide (2.1 × 150 mm, 1.7 µm) column coupled with a Waters Acquity BEH amide vanguard pre-column (2.1 × 5 mm, 1.7 µm) was used to separate carbohydrates using water with 0.1% ammonium hydroxide (A) and acetonitrile with 0.1% ammonium hydroxide (B) as the mobile phase. The following gradient was used: 0–3 min linear gradient 0–50% B, 3–6 min hold at 50% B, 6–7 min 50–0% B, and 7–12 min hold at 0% B at a constant flow rate of 0.2 mL/min and at a column temperature of 40 °C. The analysis was operated under negative electrospray ionization conditions with a TOF acquisition mass range of 150–540 m/z. Data acquisition was performed using Sciex Analyst software (version TF 1.8.1) and data analysis was performed in Sciex MultiQuant software (v 3.0). For absolute quantification, a 7-point external standard curve within a range of 2–32 µg/mL was used. Prior to LC-MS analysis, ^13^C_6_-sucrose (10 μg/mL) and ^13^C_6_-glucose (10 μg/mL) were added to each LC-MS sample as internal standards. Quantification of glucose and galactose was conducted together as the peaks of glucose and galactose did not resolve well. Thus, the areas and concentrations of each carbohydrate were added to obtain a combined standard curve. For the LC-MS analysis of glucose, fructose, galactose, and raffinose, the extracts were diluted twenty-fold. Since the concentration of sucrose was higher compared to the other carbohydrates, the analysis of sucrose was performed separately on a 1500-fold diluted extract using the same LC-MS method described above.

### 4.6. Statistical Analysis

Statistical analysis was performed using ‘R’ (https://www.R-project.org, accessed on 27 January 2024). Statistical significance reported for any analysis is defined as *p* < 0.05.

### 4.7. Data Availability

The raw data resulting from the microbiome sequencing (BioProject ID: PRJNA1163729) were submitted to the NCBI SRA database.

## 5. Conclusions

Through this work, we demonstrate sugar beet genotype-specific root microbiome-related traits that potentially play an important role in root resistance against pathogens during prolonged post-harvest indoor storage. We further demonstrate the putative role of root metabolites (e.g., N-containing SMs) that may control relative abundances of specific beneficial microbes that potentially contribute to resistance against storage pathogens. The knowledge obtained on the roles of microbiome/metabolites/genotypes in improving post-harvest storage quality will be useful for future breeding strategies to generate sugar beet cultivars with improved storage performance, thereby minimizing sugar loss and increasing profits for the growers. The prediction of microbial functions from 16S and ITS sequencing and the putative role of metabolites in improved post-harvest storage presented in this study are approximations and will require experimental validation in the future.

## Figures and Tables

**Table 1:** Alpha diversity in the resistant (R: KSG2, 3, 4, and 6) and susceptible (S: Sus_Ck) sugar beet genotypes at mid (M) and late (L) storage time points. (**A**) Bacterial diversity and (**B**) fungal diversity. The data are mean ± standard error of 4 replicates (each replicate consists of 2 sugar beet roots); Student’s *t* test: *p* < 0.05 * between the susceptible and resistant genotypes at M and L time points.

(A)		
Treatment	Observed_OTUs	Shannon
M_KSG2	186.25 ± 23.84	1.26 ± 0.09
M_KSG3	221.25 ± 17.50	1.17 ± 0.09
M_KSG4	133.5 * ± 28.12	1.04 ± 0.10
M_KSG6	257.75 ± 50.80	1.48 ± 0.25
M_Sus_Ck	214 ± 19.55	1.35 ± 0.13
L_KSG2	231 ± 26.54	1.17 ± 0.09
L_KSG3	243.75 ± 48.02	1.49 ± 0.13
L_KSG4	235.50 ± 26.22	1.21 ± 0.05
L_KSG6	273 ± 38.94	1.84 ± 0.28
L_Sus_Ck	185.50 ± 33.28	1.60 ± 0.32
**(B)**		
M_KSG2	227.75 ± 17	7.27 ± 0.11
M_KSG3	233.25 ± 19.75	7.26 ± 0.16
M_KSG4	278.25 ± 56.81	7.48 ± 0.29
M_KSG6	297.75 * ± 27.75	7.59 * ± 0.08
M_Sus_Ck	210 ± 83.85	7.08 ± 2.34
L_KSG2	180.75 ± 6.14	6.86 ± 0.13
L_KSG3	200 ± 10.40	7.04 ± 0.15
L_KSG4	223.75 ± 14.05	7.21 ± 0.09
L_KSG6	179.75 ± 7.34	6.93 ± 0.09
L_Sus_Ck	199 ± 30.11	7.04 ± 0.25

**Table 2:** Mobile phase gradient elution program for untargeted metabolomics analysis.

Time (min)	Flow Rate (mL/min)	A (%)	B (%)
0.00	0.30	95	5
1.00	0.30	95	5
12.50	0.30	5	95
13.50	0.30	5	95
13.60	0.30	95	5
16.00	0.30	95	5

## Data Availability

The authors declare availability of data and material upon request.

## Supplementary Materials

The following supporting information can be downloaded at: https://www.mdpi.com/article/10.3390/ijms252312681/s1.
