# Supplementary material for: Root Microbiome and Metabolome Traits Associated with Improved Post-Harvest Root Storage for Sugar Beet Breeding Lines Under Southern Idaho Conditions
Source: Int J Mol Sci. 2024 Nov 26;25(23):12681. doi: 10.3390/ijms252312681 (PMC11640815; doi:10.3390/ijms252312681)
Supplement: Supplementary file 1 [file ijms-25-12681-s001.zip › ijms-3257123-supplementary.pdf]

Table S1. Overview of 16S reads for different samples. Sus_Ck: susceptible genotype; KSG2, KSG3, KSG4, and KSG6: resistant genotypes; M: mid and L: late storage time points; M = million.

Sample	Raw_Tags	Raw_Bases	Valid_Tags	Valid_Bases	Valid%	Q20%	Q30%	GC%
L_KSG6_R4	85566	42.78M	79138	32.03M	92.49	95.59	87.78	56.17
L_KSG6_R3	81452	40.73M	73955	30.00M	90.80	96.61	90.62	55.92
L_KSG6_R2	85792	42.90M	79917	32.32M	93.15	96.48	90.21	55.99
L_KSG6_R1	82321	41.16M	76124	30.82M	92.47	96.23	89.51	56.00
L_KSG4_R4	81027	40.51M	75527	30.54M	93.21	96.60	90.54	55.96
L_KSG4_R3	85194	42.60M	79218	32.04M	92.99	96.60	90.57	55.94
L_KSG4_R2	84616	42.31M	78881	31.90M	93.22	96.50	90.19	56.00
L_KSG4_R1	85475	42.74M	79638	32.22M	93.17	96.68	90.74	55.96
L_KSG3_R4	81987	40.99M	76333	30.89M	93.10	96.32	89.64	56.09
L_KSG3_R3	84512	42.26M	78196	31.66M	92.53	96.74	91.02	55.92
L_KSG3_R2	86247	43.12M	80417	32.52M	93.24	96.74	90.98	55.93
L_KSG3_R1	84957	42.48M	78837	31.88M	92.80	95.80	88.37	56.06
L_KSG2_R4	85920	42.96M	80250	32.46M	93.40	96.85	91.24	55.93
L_KSG2_R3	86789	43.39M	80331	32.48M	92.56	96.79	91.15	55.96
L_KSG2_R2	81782	40.89M	76405	30.89M	93.43	96.72	91.01	55.99
L_KSG2_R1	85530	42.77M	79967	32.35M	93.50	96.90	91.42	55.98
L_Sus_Ck_R4	81443	40.72M	74822	30.26M	91.87	94.66	85.71	56.27
L_Sus_Ck_R3	85280	42.64M	80063	32.38M	93.88	96.95	91.46	55.94
L_Sus_Ck_R2	84551	42.28M	78901	31.92M	93.32	96.64	90.52	55.98
L_Sus_Ck_R1	83604	41.80M	75393	30.83M	90.18	96.02	89.71	55.03
M_KSG6_R4	80638	40.32M	74987	30.37M	92.99	96.97	91.50	55.91
M_KSG6_R3	86763	43.38M	80070	32.41M	92.29	96.93	91.40	55.96
M_KSG6_R2	82992	41.50M	77673	31.42M	93.59	96.86	91.17	55.94
M_KSG6_R1	87425	43.71M	82129	33.21M	93.94	97.04	91.75	55.92
M_KSG4_R4	83998	42.00M	77764	31.44M	92.58	95.53	87.76	56.14
M_KSG4_R3	86853	43.43M	80866	32.69M	93.11	96.57	90.64	55.98
M_KSG4_R2	80681	40.34M	74834	30.25M	92.75	96.49	90.41	55.99
M_KSG4_R1	87688	43.84M	80991	32.76M	92.36	96.47	90.38	55.99
M_KSG3_R4	81784	40.89M	76137	30.79M	93.10	96.71	90.92	55.95
M_KSG3_R3	80869	40.43M	74316	30.06M	91.90	96.69	90.95	55.95
M_KSG3_R2	81304	40.65M	75948	30.73M	93.41	96.59	90.71	55.99
M_KSG3_R1	81042	40.52M	75844	30.67M	93.59	96.72	91.01	55.97
M_KSG2_R4	82242	41.12M	75788	30.65M	92.15	95.44	87.52	56.13
M_KSG2_R3	85126	42.56M	78564	31.78M	92.29	96.45	90.15	55.97
M_KSG2_R2	81445	40.72M	75434	30.51M	92.62	96.33	89.87	55.96
M_KSG2_R1	87478	43.74M	81009	32.78M	92.60	96.40	90.07	55.94
M_Sus_Ck_R4	80626	40.31M	74899	30.29M	92.90	96.54	90.33	55.94
M_Sus_Ck_R3	82885	41.44M	75968	30.74M	91.65	96.91	91.45	55.93
M_Sus_Ck_R2	80643	40.32M	74542	30.19M	92.43	96.91	91.43	55.97
M_Sus_Ck_R1	81204	40.60M	75574	30.57M	93.07	96.96	91.54	55.95

Table S2. Overview of ITS reads for different samples. Sus_Ck: susceptible genotype; KSG2, KSG3, KSG4, and KSG6: resistant genotypes; M: mid and L: late storage time points; M = million.

Sample	Raw_Tags	Raw_Bases	Valid_Tags	Valid_Bases	Valid%	Q20%	Q30%	GC%
L_KSG6_R4	370217	185.11M	360265	99.48M	97.31	97.64	94.77	55.12
L_KSG6_R3	354174	177.09M	345373	92.18M	97.52	97.86	95.24	54.70
L_KSG6_R2	342599	171.30M	334855	92.65M	97.74	97.77	95.03	55.08
L_KSG6_R1	325407	162.70M	317592	87.56M	97.60	97.34	93.51	55.14
L_KSG4_R4	349380	174.69M	341058	94.23M	97.62	97.66	94.81	55.03
L_KSG4_R3	422970	211.49M	413287	114.64M	97.71	97.77	95.00	55.12
L_KSG4_R2	413507	206.75M	403237	111.90M	97.52	97.52	94.53	55.24
L_KSG4_R1	462692	231.35M	451937	125.20M	97.68	97.21	93.08	55.22
L_KSG3_R4	423875	211.94M	410345	105.75M	96.81	98.04	95.64	53.32
L_KSG3_R3	428632	214.32M	418594	113.66M	97.66	97.88	95.28	54.62
L_KSG3_R2	351907	175.95M	344081	95.47M	97.78	97.84	95.17	55.10
L_KSG3_R1	302691	151.35M	294909	81.89M	97.43	96.85	91.71	55.28
L_KSG2_R4	355025	177.51M	346899	95.87M	97.71	97.74	94.96	55.04
L_KSG2_R3	340110	170.06M	332202	91.83M	97.67	97.82	95.14	55.05
L_KSG2_R2	321152	160.58M	313307	82.79M	97.56	97.95	95.47	55.05
L_KSG2_R1	378639	189.32M	369547	100.59M	97.60	97.33	93.31	54.81
L_Sus_Ck_R4	467122	233.56M	455478	122.80M	97.51	97.87	95.26	54.66
L_Sus_Ck_R3	506175	253.09M	494813	126.99M	97.76	98.40	96.39	52.76
L_Sus_Ck_R2	8137	4.07M	7227	2.00M	88.82	97.66	94.67	55.10
L_Sus_Ck_R1	361152	180.58M	353015	88.02M	97.75	97.98	94.97	52.51
M_KSG6_R4	509600	254.80M	498311	136.75M	97.78	97.77	95.04	54.96
M_KSG6_R3	440152	220.08M	430347	119.03M	97.77	97.79	95.01	55.05
M_KSG6_R2	434844	217.42M	424691	117.57M	97.67	97.66	94.85	55.17
M_KSG6_R1	543608	271.80M	530625	147.34M	97.61	97.01	92.28	55.26
M_KSG4_R4	418540	209.27M	407686	113.18M	97.41	97.69	94.90	55.16
M_KSG4_R3	802152	401.08M	783777	217.70M	97.71	97.76	95.04	55.13
M_KSG4_R2	428458	214.23M	418786	116.24M	97.74	97.80	95.08	55.10
M_KSG4_R1	377397	188.70M	368467	101.56M	97.63	97.25	93.12	55.12
M_KSG3_R4	408270	204.13M	398812	110.56M	97.68	97.70	94.90	55.09
M_KSG3_R3	491256	245.63M	480225	133.01M	97.75	97.78	95.00	55.07
M_KSG3_R2	394254	197.13M	384532	106.65M	97.53	97.62	94.79	55.19
M_KSG3_R1	358918	179.46M	350249	97.21M	97.58	97.21	93.11	55.24
M_KSG2_R4	348872	174.44M	340233	94.02M	97.52	97.64	94.78	55.14
M_KSG2_R3	399074	199.54M	390126	107.32M	97.76	97.76	95.03	55.03
M_KSG2_R2	479042	239.52M	469074	130.07M	97.92	97.77	95.04	55.12
M_KSG2_R1	466371	233.19M	456346	126.33M	97.85	97.37	93.64	55.18

M_Sus_Ck_R4	7218	3.61M	6492	1.80M	89.94	97.58	94.54	55.15
M_Sus_Ck_R3	9934	4.97M	9137	2.52M	91.98	97.73	94.92	55.05
M_Sus_Ck_R2	402605	201.30M	393395	107.88M	97.71	97.66	94.88	55.07
M_Sus_Ck_R1	444288	222.14M	434466	119.76M	97.79	97.31	93.42	55.16

Table S3. Percent abundance of bacterial phyla in sugar beet roots across all treatments. Sus_Ck: susceptible genotype; KSG2, KSG3, KSG4, and KSG6: resistant genotypes; M: mid and L: late storage time points. Data are mean of 4 replicates (each replicate consists of samples obtained from two roots).

Phylum	L_KSG6	L_KSG4	L_KSG3	L_KSG2	L_Sus_Ck	M_KSG6	M_KSG4	M_KSG3	M_KSG2	M_Sus_Ck
Cyanobacteria	75.9747	82.7136	75.6933	83.0837	75.9888	79.3585	82.2704	83.7597	77.9862	80.2222
Proteobacteria	15.4526	12.8985	19.0340	12.7058	19.1161	14.4167	15.4797	12.6597	18.4049	13.9985
Actinobacteriota	6.4460	2.7446	3.8347	2.9944	3.6277	4.4875	1.3227	2.1865	2.1066	3.8077
Firmicutes	1.0510	0.8259	0.5718	0.7041	0.8802	1.2237	0.5337	0.7665	0.7693	1.2828
Bacteroidota	0.4401	0.1593	0.2069	0.1441	0.0766	0.1299	0.0776	0.1594	0.1562	0.1086
Verrucomicrobiota	0.2160	0.1494	0.1528	0.0636	0.1021	0.0650	0.1091	0.0972	0.0921	0.0440
Chloroflexi	0.0911	0.0927	0.1167	0.0838	0.0532	0.0875	0.0369	0.0857	0.0574	0.0551
Desulfobacterota	0.0169	0.0243	0.0021	0.0046	0.0000	0.0022	0.0469	0.0685	0.2558	0.2978
Planctomycetota	0.1384	0.1132	0.1308	0.0408	0.0530	0.0598	0.0418	0.0452	0.0320	0.0323
Acidobacteriota	0.0358	0.1008	0.1074	0.0573	0.0371	0.0718	0.0268	0.0606	0.0405	0.0545
Myxococcota	0.0500	0.0920	0.0980	0.0475	0.0262	0.0501	0.0276	0.0774	0.0485	0.0401
Patescibacteria	0.0248	0.0322	0.0226	0.0456	0.0252	0.0275	0.0025	0.0073	0.0212	0.0012
Gemmatimonadota	0.0030	0.0105	0.0094	0.0115	0.0039	0.0072	0.0115	0.0072	0.0048	0.0185
Deinococcota	0.0305	0.0008	0.0041	0.0054	0.0073	0.0027	0.0058	0.0033	0.0000	0.0114
Fusobacteriota	0.0017	0.0051	0.0000	0.0015	0.0000	0.0046	0.0000	0.0048	0.0127	0.0129
unclassified	0.0128	0.0174	0.0016	0.0000	0.0000	0.0000	0.0008	0.0008	0.0000	0.0000
Synergistota	0.0073	0.0113	0.0015	0.0000	0.0012	0.0000	0.0000	0.0016	0.0000	0.0000

Bdellovibrionota	0.0008	0.0016	0.0029	0.0000	0.0000	0.0018	0.0023	0.0033	0.0008	0.0012
Deferribacterota	0.0000	0.0000	0.0000	0.0000	0.0000	0.0000	0.0000	0.0000	0.0073	0.0073
Armatimonadota	0.0008	0.0000	0.0051	0.0000	0.0000	0.0007	0.0000	0.0008	0.0023	0.0008
Campylobacterota	0.0000	0.0000	0.0012	0.0016	0.0000	0.0000	0.0017	0.0045	0.0000	0.0008
Nitrospirota	0.0021	0.0023	0.0015	0.0008	0.0000	0.0015	0.0008	0.0000	0.0000	0.0000
Elusimicrobiota	0.0000	0.0020	0.0009	0.0012	0.0000	0.0008	0.0008	0.0000	0.0000	0.0000
Abditibacteriota	0.0000	0.0012	0.0008	0.0011	0.0000	0.0000	0.0000	0.0000	0.0000	0.0012
Sumerlaeota	0.0029	0.0000	0.0000	0.0000	0.0000	0.0000	0.0008	0.0000	0.0000	0.0000
Candidatus_Saccharibacteria	0.0000	0.0000	0.0000	0.0000	0.0015	0.0000	0.0000	0.0000	0.0016	0.0000
Methylomirabilota	0.0000	0.0000	0.0000	0.0019	0.0000	0.0000	0.0000	0.0000	0.0000	0.0000
Fibrobacterota	0.0000	0.0015	0.0000	0.0000	0.0000	0.0000	0.0000	0.0000	0.0000	0.0000
WPS-2	0.0000	0.0000	0.0000	0.0000	0.0000	0.0000	0.0000	0.0000	0.0000	0.0012
Spirochaetota	0.0008	0.0000	0.0000	0.0000	0.0000	0.0000	0.0000	0.0000	0.0000	0.0000
Others	0.0000	0.0000	0.0000	0.0000	0.0000	0.0008	0.0000	0.0000	0.0000	0.0000

Table S4. Relative abundance of significantly altered bacterial phyla at late storage time point (L). Data are mean of 4 replicates (each replicate consists of samples obtained from two roots); susceptible genotype: Sus_Ck and resistant genotypes: KSG2, KSG3, KSG4, and KSG6; g: genus.

Genus	kruskal.test.p_value	significance	mean_L_Sus_Ck	mean_L_KSG2	mean_L_KSG3	mean_L_KSG4	mean_L_KSG6
g__Flavonifractor	0.00	yes	0.000	0.015	0.013	0.000	0.000
g__unclassified	0.00	yes	0.000	0.000	0.002	0.017	0.013
g__Brachybacterium	0.00	yes	0.092	0.026	0.021	0.002	0.319
g__Faecalibacterium	0.00	yes	0.020	0.040	0.031	0.049	0.058
g__Desulfovibrio	0.00	yes	0.001	0.003	0.002	0.024	0.017
g__Saccharofermentans	0.00	yes	0.000	0.006	0.008	0.000	0.000
g__Tuzzerella	0.01	yes	0.000	0.000	0.000	0.012	0.005
g__Rikenellaceae_RC9_gut_group	0.01	yes	0.000	0.001	0.000	0.026	0.016
g__Ralstonia	0.01	yes	0.217	0.152	0.116	0.116	0.096
g__Turicibacter	0.01	yes	0.000	0.000	0.000	0.011	0.004
g__Verrucomicrobiota_unclassified	0.01	yes	0.007	0.000	0.000	0.000	0.000
g__Georgenia	0.01	yes	0.000	0.000	0.000	0.000	0.020
g__Chitinophagaceae_unclassified	0.01	yes	0.000	0.000	0.000	0.000	0.011
g__Tundrisphaera	0.01	yes	0.000	0.000	0.000	0.000	0.007
g__Lawsonella	0.01	yes	0.003	0.000	0.000	0.000	0.000
g__Sanguibacter-Flavimobilis	0.01	yes	0.017	0.002	0.002	0.000	0.090
g__Terribacillus	0.01	yes	0.051	0.000	0.003	0.002	0.001
g__Clostridiales_unclassified	0.01	yes	0.009	0.002	0.009	0.022	0.042
g__Lactobacillus	0.01	yes	0.140	0.092	0.082	0.075	0.053
g__Marmoricola	0.01	yes	0.159	0.038	0.033	0.013	0.078
g__Moraxellaceae_unclassified	0.02	yes	0.000	0.015	0.008	0.004	0.000
g__Escherichia-Shigella	0.02	yes	0.000	0.015	0.015	0.006	0.010
g__Xanthobacteraceae_unclassified	0.02	yes	0.003	0.000	0.013	0.003	0.000
g__Sinobaca	0.02	yes	0.022	0.022	0.009	0.008	0.203
g__Clostridium	0.02	yes	0.008	0.004	0.003	0.039	0.014
g__Erythrobacteraceae_unclassified	0.02	yes	0.000	0.068	0.071	0.045	0.034
g__Vicinamibacteraceae_unclassified	0.02	yes	0.000	0.011	0.007	0.025	0.000
g__Candidatus_Saccharimonas	0.02	yes	0.020	0.021	0.011	0.002	0.000
g__Microtrichales_unclassified	0.03	yes	0.000	0.002	0.008	0.008	0.001
g__Streptococcus	0.03	yes	0.006	0.017	0.019	0.000	0.002
g__Polaromonas	0.03	yes	0.000	0.011	0.000	0.012	0.005
g__Carnobacterium	0.03	yes	0.000	0.059	0.001	0.000	0.000
g__Microvirga	0.03	yes	0.000	0.002	0.008	0.002	0.018
g__Fimbriimonadaceae_unclassified	0.03	yes	0.000	0.000	0.005	0.000	0.001
g__Nesterenkonia	0.03	yes	0.068	0.039	0.043	0.037	0.393
g__Eubacterium]_coprostanoligenes_group_unclassified	0.03	yes	0.005	0.004	0.007	0.020	0.015
g__Chloroplast_unclassified	0.03	yes	75.989	83.083	75.689	82.711	75.975
g__Cellulomonas	0.03	yes	0.004	0.004	0.021	0.007	0.035

g__Knoellia	0.03	yes	0.022	0.010	0.000	0.004	0.059
g__Pseudoclavibacter	0.03	yes	0.020	0.000	0.002	0.000	0.041
g__Ligilactobacillus	0.03	yes	0.066	0.012	0.024	0.007	0.022
g__Paenarthrobacter	0.04	yes	0.098	0.038	0.083	0.051	0.372
g__Erwinia	0.04	yes	0.007	0.000	0.000	0.000	0.141
g__Olsenella	0.04	yes	0.000	0.000	0.000	0.010	0.003
g__Amaricoccus	0.04	yes	0.000	0.000	0.000	0.001	0.005
g__Megasphaera	0.04	yes	0.000	0.000	0.000	0.009	0.003
g__UCG-010_unclassified	0.04	yes	0.003	0.000	0.000	0.000	0.001
g__Aeromicrobium	0.04	yes	0.125	0.113	0.094	0.045	0.324
g__Pseudonocardia	0.04	yes	0.155	0.045	0.063	0.055	0.200
g__Kocuria	0.05	yes	0.055	0.009	0.025	0.031	0.078
g__Hyphomicrobiaceae_unclassified	0.05	yes	0.000	0.003	0.007	0.000	0.010
g__Muribaculaceae_unclassified	0.05	yes	0.021	0.047	0.020	0.020	0.013
g__Lysinimonas	0.05	yes	0.000	0.026	0.031	0.000	0.024
g__Butyricicoccus	0.05	yes	0.000	0.005	0.008	0.000	0.000
g__Gaiella	0.05	yes	0.000	0.005	0.014	0.004	0.003
g__Blautia	0.05	yes	0.008	0.005	0.001	0.005	0.000

Table S5. Percent abundance of fungal phyla in sugar beet roots across all treatments. Sus_Ck: susceptible genotype; KSG2, KSG3, KSG4, and KSG6: resistant genotypes; M: mid and L: late storage time points. Data are mean of 4 replicates (each replicate consists of samples obtained from two roots).

**Phylum**	**L_KSG6**	**L_KSG4**	**L_KSG3**	**L_KSG2**	**L_Sus_Ck**	**M_KSG6**	**M_KSG4**	**M_KSG3**	**M_KSG2**	**M_Sus_Ck**
Fungi_unclassified	84.012	83.508	83.719	85.419	78.167	84.438	85.095	84.344	85.281	79.346
Ascomycota	15.956	16.450	16.275	14.493	21.087	15.559	14.903	15.647	14.706	20.603
Basidiomycota	0.032	0.043	0.006	0.087	0.744	0.003	0.002	0.009	0.012	0.052
Zygomycota	0.000	0.000	0.000	0.000	0.002	0.000	0.000	0.000	0.001	0.000

Table S6. Root surface area coverage with disease symptoms estimated at late time point of storage. Sus_Ck: susceptible genotype; KSG2, KSG3, KSG4, and KSG6: resistant genotypes. Data are mean of 10 biological replicates; $P < 0.05$* between the susceptible (Sus_Ck) and resistant genotypes (KSG2, KSG3, KSG4, and KSG6).

Genotype	% surface disease coverage
Sus_Ck	18
KSG2	5*
KSG3	3*
KSG4	2*
KSG6	2*

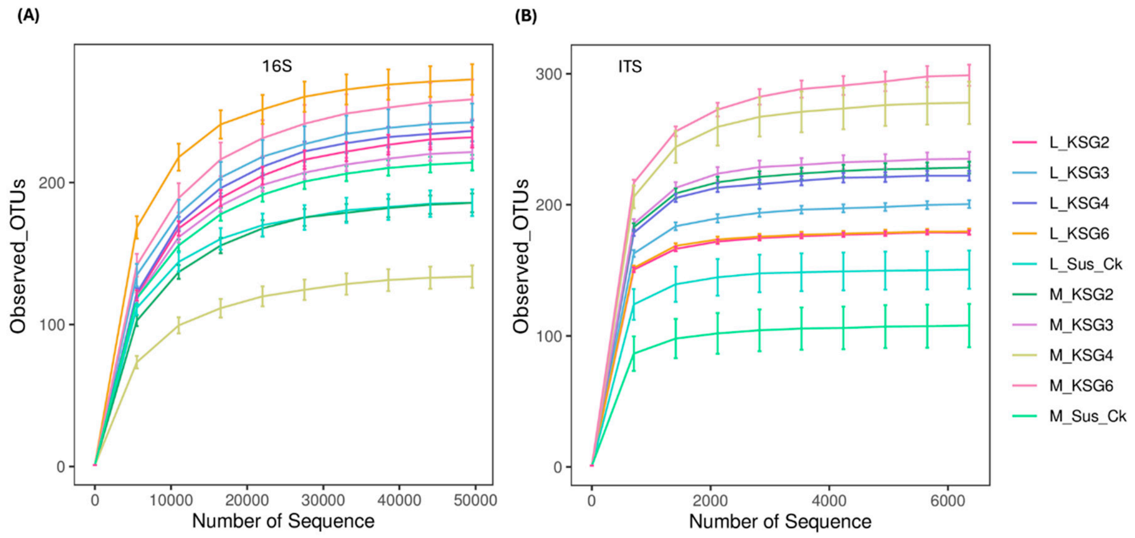

Figure S1. The rarefaction curves obtained from all samples sequenced. (A) 16S; and (B) ITS. Sus.Ck: susceptible genotype; KSG2, KSG3, KSG4, and KSG6: relatively resistant genotypes; M: mid and L: late storage time points; OTU: operational taxonomic unit.

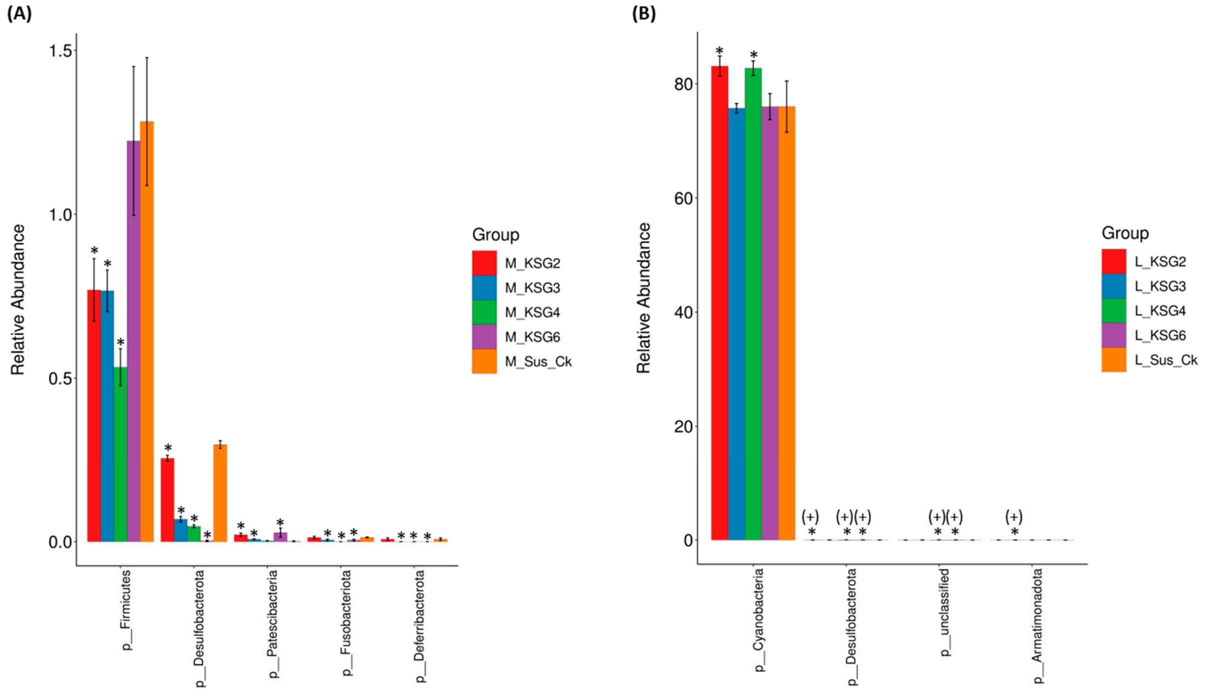

Figure S2. Relative abundance of significantly altered bacterial phyla at (A) mid; and (B) late storage time points. Data are mean ± standard error of 4 replicates (each replicate consists of samples obtained from two roots); $P < 0.05^*$ between the susceptible (Sus_Ck) and resistant genotypes (KSG2, KSG3, KSG4, and KSG6) within specific storage time points, mid (M) and late (L); (+) denotes increase; p: phylum.

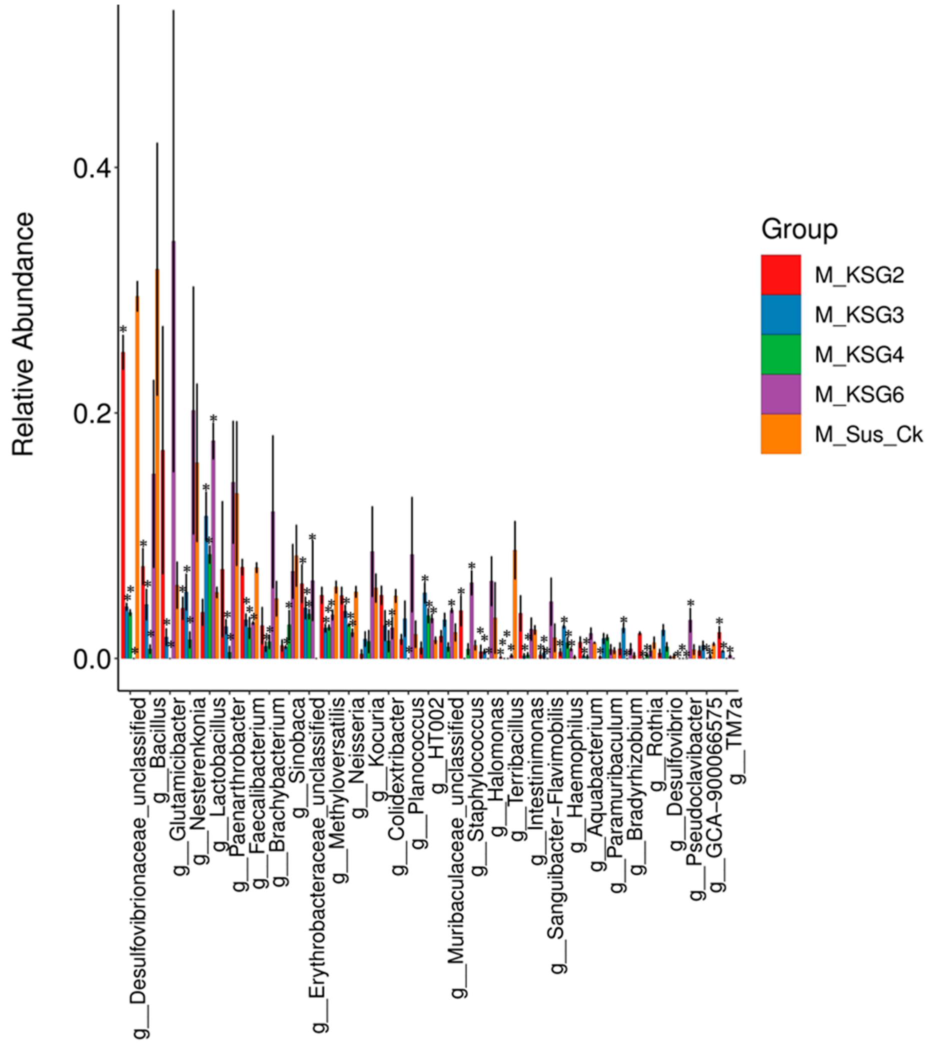

Figure S3. Relative abundance of significantly altered bacterial genera at mid storage time point (M). Data are mean ± standard error of 4 replicates (each replicate consists of samples obtained from two roots); $P < 0.05$* between the susceptible (Sus_Ck) and resistant genotypes (KSG2, KSG3, KSG4, and KSG6); g: genus.

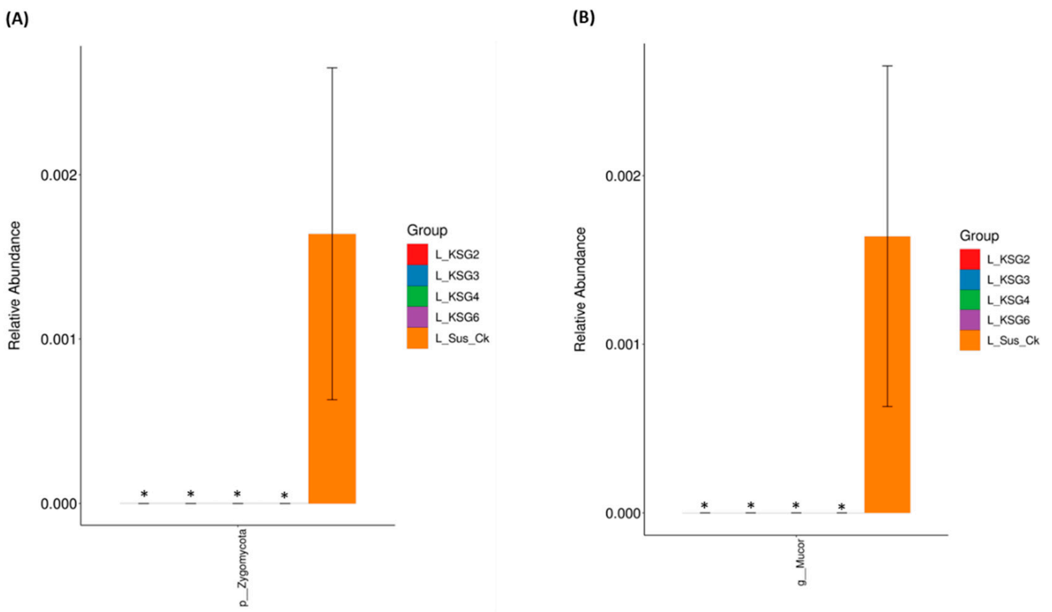

Figure S4. Relative abundance of significantly altered fungal (A) phylum; and (B) genus at late (L) storage time point. Data are mean ± standard error of 4 replicates (each replicate consists of samples obtained from two roots); $P < 0.05$* between the susceptible (Sus_Ck) and resistant genotypes (KSG2, KSG3, KSG4, and KSG6); p: phylum and g: genus.

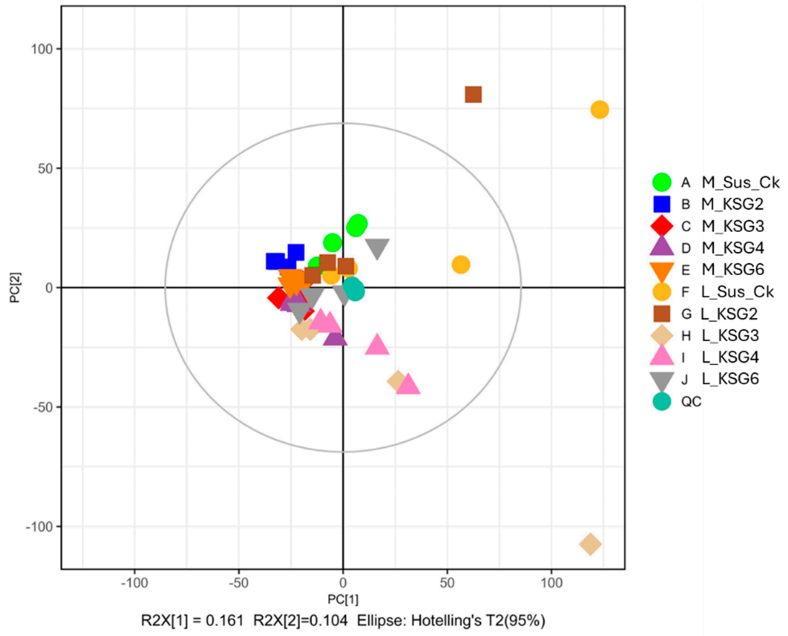

Figure S5. Principal coordinate analysis (PCoA) of fungal diversity of untargeted metabolome data originating from the samples. Sus.Ck: susceptible genotype; KSG2, KSG3, KSG4, and KSG6: relatively resistant genotypes; M: mid and L: late storage time points; QC: quality control.

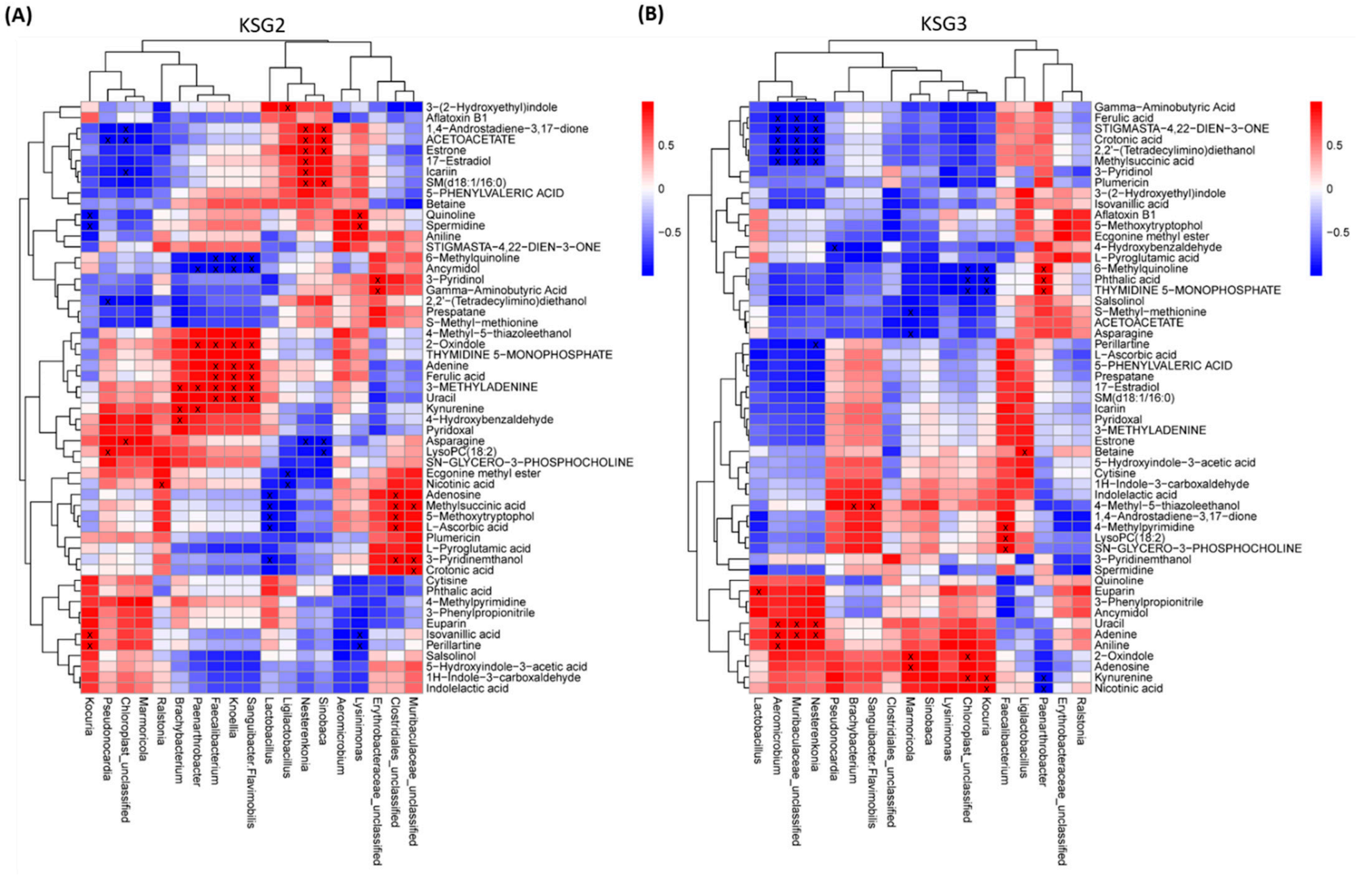

Figure S6. Correlation analysis between root bacteriome and metabolome at late storage time point reveals distinct pattern in the resistant genotypes vs. susceptible genotype. (A) Resistant genotype, KSG2; and (B) resistant genotype, KSG3. Data are mean ± standard error of 4 replicates (each replicate consists of samples obtained from two roots). An ‘X’ sign inside the rectangular boxes in the heatmap indicates $P < 0.05$.

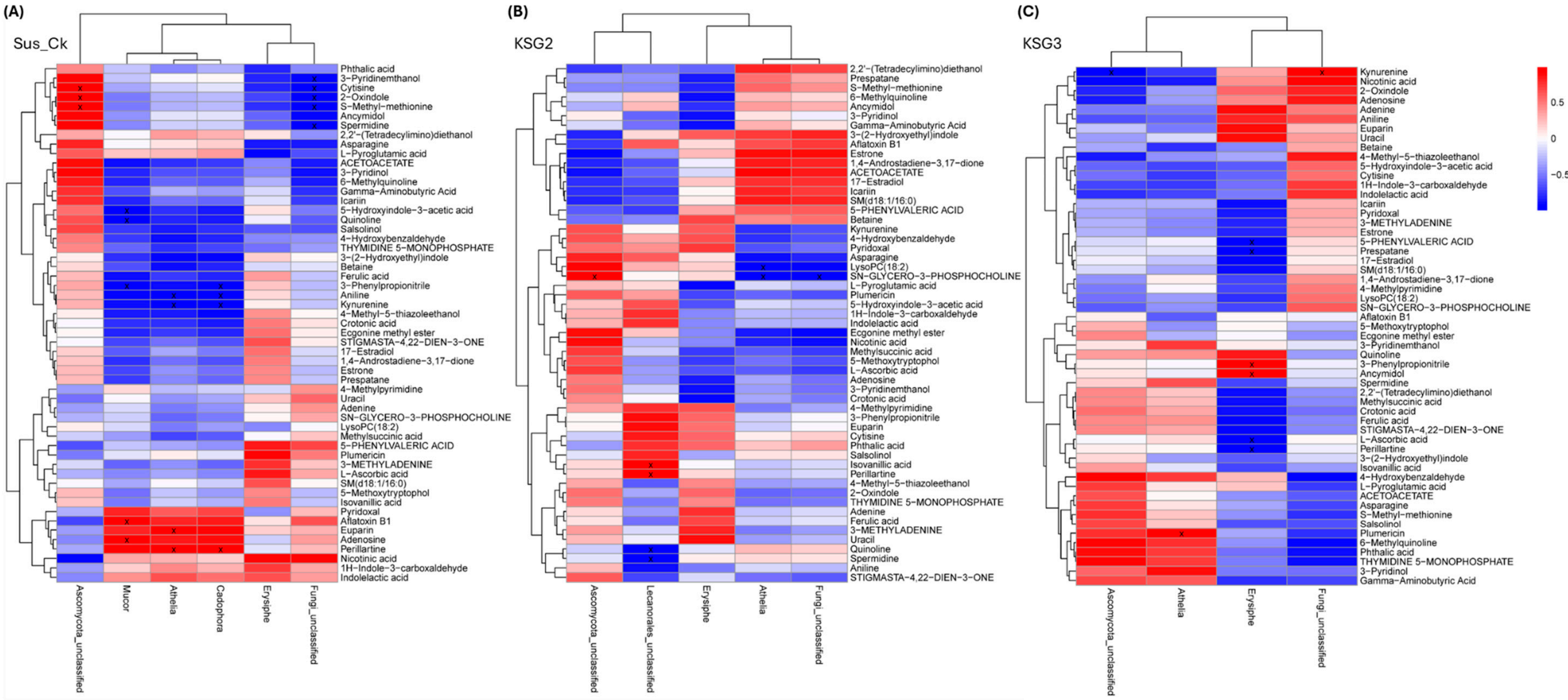

Figure S7 (continued)

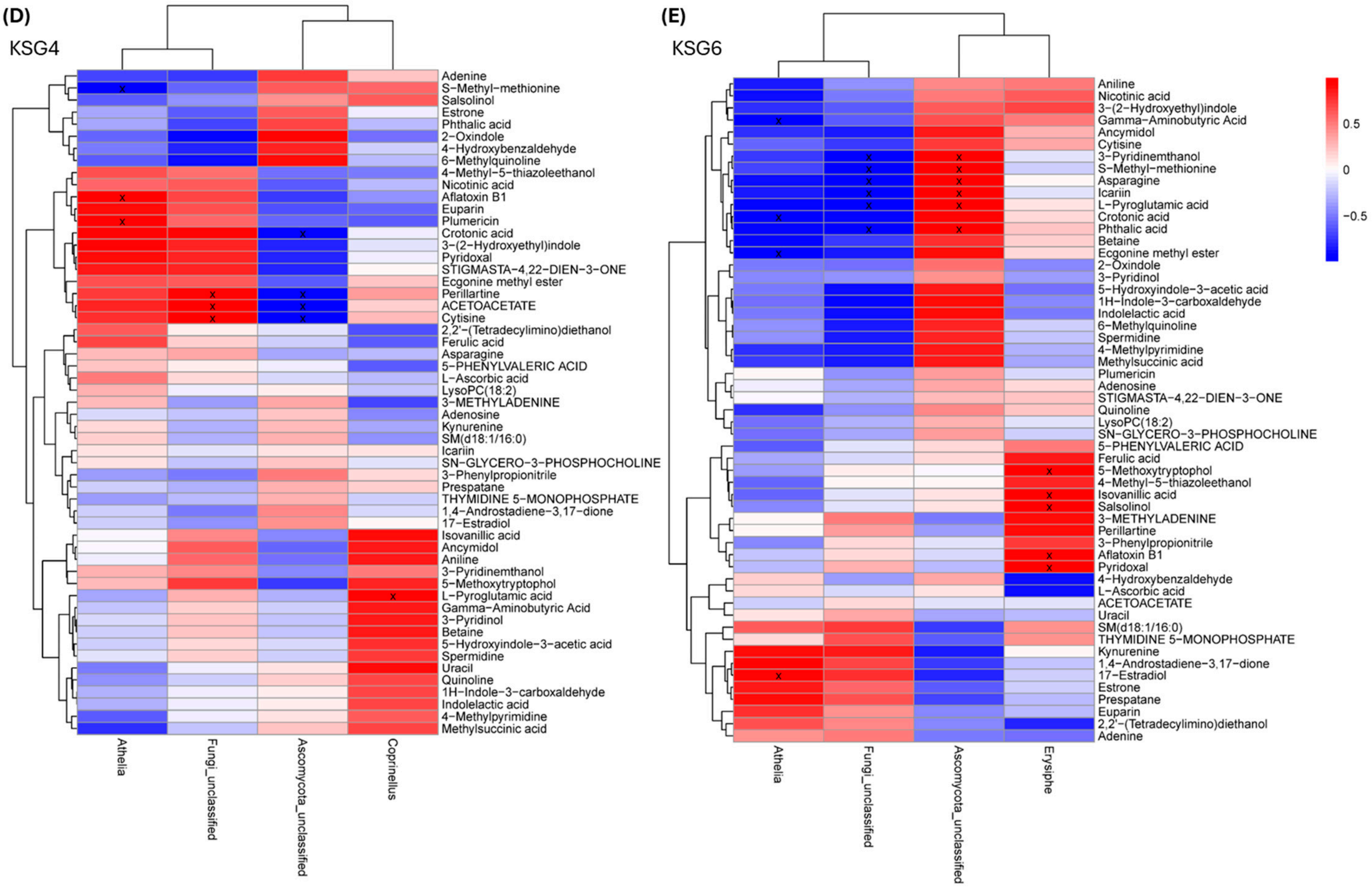

Figure S7. Correlation analysis between root fungal microbiome and metabolome at late storage time point reveals distinct pattern in the susceptible vs. resistant genotypes. (A) Susceptible genotype, Sus_Ck; (B) resistant genotype, KSG2; (C) resistant genotype, KSG3; (D) resistant genotype, KSG4; and (E) resistant genotype, KSG6. Data are mean ± standard error of 4 replicates (each replicate consists of samples obtained from two roots). An ‘X’ sign inside the rectangular boxes in the heatmap indicates $P < 0.05$.

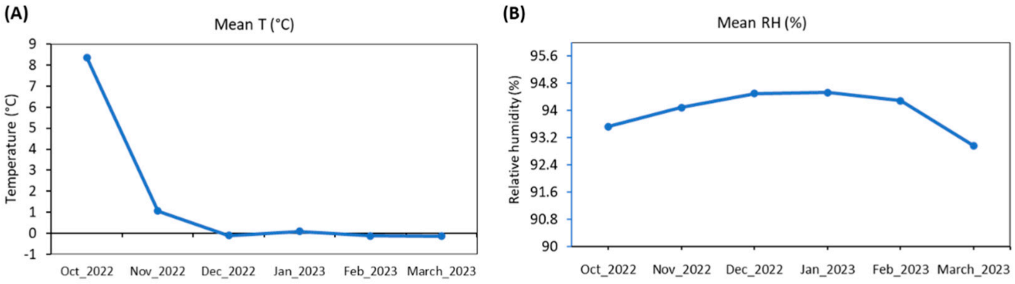

Figure S8. Mean (A) temperature (T); and (B) relative humidity (RH) inside the indoor sugar beet storage building [located at Paul, ID (USA)] from mid-October through mid-March. Temperature and RH data were collected hourly for each day of the month.
